# The impact of articulatory consciousness training on reading and spelling literacy in students with severe dyslexia: an experimental single case study

**DOI:** 10.1007/s11881-021-00225-1

**Published:** 2021-04-30

**Authors:** Anne Cathrine Thurmann-Moe, Monica Melby-Lervåg, Arne Lervåg

**Affiliations:** 1 Department of Special Needs Education and Statped (National Service for Special Needs Education), University of Oslo, Oslo, Norway; 2 Department of Special Needs Education, University of Oslo, Oslo, Norway; 3 Department of Education, University of Oslo, Oslo, Norway

**Keywords:** Articulatory consciousness, Dyslexia, Severe reading disorders, Single case design

## Abstract

This study evaluates the effect of an intervention whose aim is to make articulatory consciousness a tool in decoding and spelling. The sample comprises 11 students with severe dyslexia (2 *SD* below the mean pseudoword scores), and the intervention programme consists of 32 individual sessions over 8 weeks. The study applies a multiple baseline/probe design with five baseline tests that correspond to a control condition, eight tests during the intervention and five post-intervention tests. On average, the results show significant improvement in all reading and spelling outcomes. However, there were also significant effects on an irrelevant control task (the pegboard test), perhaps indicating testing effects on the dependent variables, making it difficult to draw firm conclusions from the study. Consequently, testing the intervention in randomised trials of children with severe dyslexia is recommended to draw more firm conclusions about its efficacy for this group.

**Supplementary Information:**

The online version contains supplementary material available at 10.1007/s11881-021-00225-1.

Dyslexia is defined as a specific disorder of reading and spelling, which is primarily caused by a deficit in the phonological system (Lyon et al., 2003). The aetiology of this deficit is not fully known, but both genetic factors and environment play a role (Byrne et al., 2006; Christopher et al., 2013; Elwér et al., 2013; Olson et al., 2011). Thus, there is a large heterogeneity in children with dyslexia in terms of severity and the impact that the reading disorder has on reading, spelling and academic performance in general (Snowling & Melby-Lervåg, 2016). The heterogeneity within dyslexic samples also actualises the need for fine-grained measurement tools in the process of diagnosis and a wider spectrum of tailored remediation programmes. In this multiple probe study of students with severe dyslexia, we evaluate the effect of an instructional programme whose aim is to teach students how to use knowledge about articulatory and acoustic features of speech sounds as a tool in spelling and decoding.

## The phonological deficit hypothesis versus the multifactorial model

According to the phonological deficit hypothesis, reading and spelling problems in dyslexia are caused by underspecified phonological representations (Swan & Goswami, 1997). The weak phonological representations have often been explained with deficits in the auditory sensory system (Hämäläinen et al., 2013) or from deviant auditory perception (McBride-Chang, 1996; Noordenbos & Serniclaes, 2015).

However, more recently, the phonological deficit hypothesis has been criticised as overly simplistic, with the claim that dyslexia is not likely to be due to a single cause. This has been supported by studies showing that not all children with severe reading problems have a phonological deficit (Ramus et al., 2013).

As phonological deficit theory has not been able to accommodate important findings in children with dyslexia, researchers have suggested a multifactorial model for explaining reading disorders (Pennington et al., 2012). Recent longitudinal studies have also demonstrated that dyslexia does not stem exclusively from phonological deficits and have focused on a wider spectrum of sensory, cognitive and environmental factors (Eklund et al., 2015; Snowling et al., 2003).

## Speech production deficits in dyslexia

In addition, it has been suggested that speech perception is causally related to dyslexia (Hulme & Snowling, 2009), and that the quality of phonological representations can be understood in the context of speech production skills (Elbro et al., 1998; Snowling et al., 1992). Elbro et al. (1998) found that the distinctness of phonological representations, measured by a task where preschool children corrected a hand-held toy which incorrectly pronounced target words, correlated with later decoding skills. In a case study of a boy with speech sound disorders, Snowling et al. (1992) reported that as time went on, the boy’s speech sound problems were resolved, but the same error typology remained as a spelling problem.

Correlations between speech production deficits and reading impairment are frequently reported in samples with speech sound disorders (Lewis et al., 2011; Preston et al., 2013) and are also present in dyslexic samples (Lalain et al., 2003; Sénéchal et al., 2004). Studies also show that individuals with dyslexia perform more poorly than controls both in articulatory awareness (Griffiths & Frith, 2002; Montgomery, 1981) and articulatory speed (Duranovic & Sehic, 2013; Fawcett & Nicolson, 2002). However, studies employing a longitudinal perspective have shown that speech production skills do not seem to have unique explanatory value regarding the development of reading disorders (Hulme et al., 2015). Rather, it appears that speech production and speech perception are closely linked (Hulme & Snowling, 2009). This is perhaps most clearly formulated in the motor theory of speech perception (Liberman & Mattingly, 1985; Liberman & Whalen, 2000). According to this theory, speech perception and speech production cannot be separated; they are parts of the same process, where the perception of sounds is synchronised with the observation of associated articulatory gestures.

## Articulatory consciousness training to ameliorate dyslexia

Based on the theory that dyslexia can, at least partly, be caused by speech production problems, the next step was to try to train students in features related to speech production to ascertain whether this can enhance decoding and spelling. An instructional focus on articulation is also embedded in most basic reading programmes through read-aloud exercises. Moreover, teachers working in first-grade classrooms often report that novice readers spontaneously use loud or silent articulation as a form of support when working with segmentation and spelling tasks. This indicates that articulation may function as a ‘natural’ tool in reading and spelling (Skjelfjord, 1987). According to the self-teaching hypothesis, silent articulation may also play a role in the spontaneous process of phonological recoding (print-to-sound translation) that occurs during text reading (Share, 1999).

Interventions using articulatory training have mainly been conducted on typically developing preschool children and novice typical readers (Boyer & Ehri, 2011; Castiglioni-Spalten & Ehri, 2003; Fälth et al., 2017; Torgesen et al., 2001). In reading-delayed samples, the most frequently studied articulatory intervention programme was the Auditory Discrimination in Depth (ADD) and a later version of the same programme called LIPs (Lindamood Phonemic Sequences) (Lindamood & Lindamood, 1998; What Works Clearinghouse (WWC), 2008, 2015). This programme applies a multisensory approach to reading and aims to teach children to identify the mouth movements involved in the production of speech sounds. Several studies have compared the effects of this programme with other phonic-based programmes. Some studies of the ADD/LIP programmes have failed to demonstrate clear advantages of articulatory training over more traditional phonic approaches in improving phonological awareness and basic decoding skills, concluding that the two approaches provide similar gains (Torgesen et al., 2010; Wise et al., 1999). However, other studies have reported significant advantages of articulatory training compared to traditional phonics instruction in samples with reading problems (Joly-Pottuz et al., 2008; Trainin et al., 2014), particularly for those with the most severe reading problems (Fälth et al., 2017; Trainin et al., 2014).

In clinical contexts, a system based on pictographic symbols of both articulatory and accoustic features of the speech sounds, Pictographic Articulatory System (PAS)(Kausrud,^2003^) has been used on children with language disorders. The results of a case study of an 8-year-old boy with developmental language disorder suggest that a combined intervention, using both a semantic graphical language system, ‘Blissymbolics’ https://www.blissymbolics.org/ and PAS symbols, played a compensatory and mediating role by ameliorating the child’s ability to read (Ottem & Kausrud, 2001).

In a recent randomised controlled trial, Authors (Thurmann-Moe, Melby-Lervåg, & Lervåg, 2021) examined the effects of the PAS material in a 5-week intervention aimed at improving phonological awareness and basic decoding skills in a sample of reading-delayed (approximately below the 20 percentile) first graders ( N = 129). For this group of delayed children, no effect was found beyond a ‘business as usal’ control group using ordinary phonics i.e. linking letters to sounds based on listening, and phonological awareness training based on listening skills. However, as most of these children were beginning readers with a delay, and not dyslexic readers, we could not rule out that this kind of intervention would not work on children and young people with more severe dyslexic problems.

## The current study

Based on prior research indicating that articulatory consciousness training would produce benefits particularly in those with severe reading disorders, the current study examines the effects of an intervention in a sample of children with persistent dyslexia. The intervention material is predominantly the same as in our previous study.

Students with developmental dyslexia is a heterogeneous group, but typically it refers to the 7–10% weakest decoders (Hulme & Snowling, 2016). However, when children get older, the symptoms change and the reading problems typically are not so severe, but the problems may persist in spelling. Even if the prevalence of dyslexia is relatively high, students with more severe dyslexia are rare. Both for ethical and practical reasons, it was considered difficult to recruit equivalent participants to a control group. We therefore used a single-case experimental design (SCED) (Gast & Ledford, 2014; Tate et al., 2016). The basic logic of SCED is to compare each participant with himself by contrasting the mean level of performance from repeated measures in the phase prior to intervention (baseline) with the mean level of performance after intervention onset (intervention and post phase). SCED has the potential to achieve experimental control, and are considered ‘true experiments’, according to current evidence standards (Cook et al., 2015; Kratochwill et al., 2010, 2013; Tate et al., 2016). SCED are particularly appropriate for pilot work prior to larger experiments, and to examine intervention effects in marginalised groups within the field of special education (Gast & Ledford, 2014; Horner et al., 2005; Lobo et al., 2017; Shadish, 2014; Shadish et al., 2015).

The hypothesis underlying the intervention is that increased articulatory consciousness makes the discrimination of speech sounds easier for individuals with dyslexia and, therefore, makes the representations of phonemes in the memory more distinct, producing a sustained training effect.

The research questions for the study are as follows:

- Will articulatory consciousness training improve the students’ reading efficiency and reading accuracy concerning regular words, pseudowords and irregular words?
- Will articulatory consciousness training improve the students’ spelling efficiency?

## Method

### Sample

**Fig. 1:**
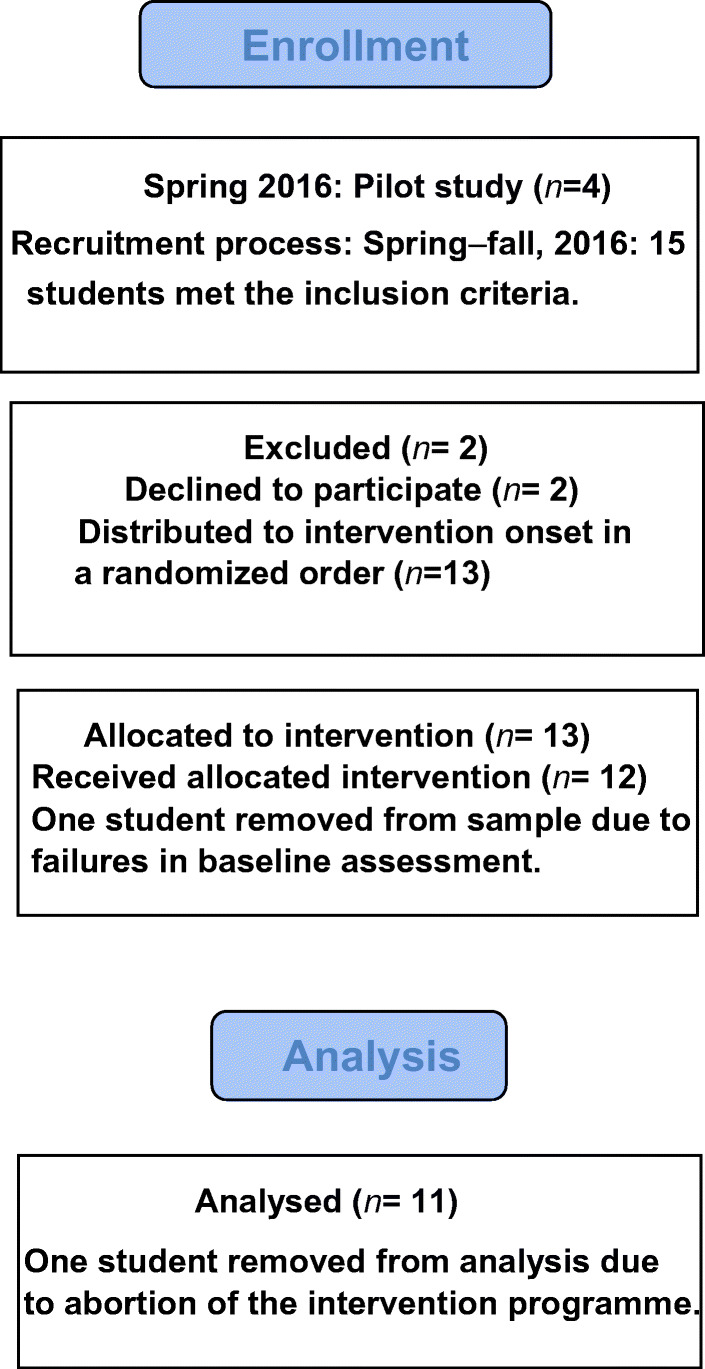
Flow of participants

The sample was recruited from the Regional Department for Speech and Language Disorders at The National Service for Special Needs Education. All students were referred due to persistent dyslexia. Further, criteria for participating in the study were scores below 2 *SD* on two standardised pseudo-reading subtests (STAS, Klinkenberg & Skaar, 2003), i.e. below the second percentile; the participants’ first language should be Norwegian; and they should be aged 10 years or older. Students with more complex diagnoses were excluded. For the flow of participants through the study, see Fig. 1.

Additional sample characteristics were collected on a standardised pseudoword spelling test, also from the STAS battery, and on selected subtests from the Norwegian standardised version of the WISC-IV (Wechsler, 2003). The students’ parents also answered a questionnaire concerning their children’s early development, family risk factors and socio-economic status. For further details concerning the sample descriptives, see Table 1.

**Table 1:** Sample descriptives

						Scores						
Case characteristics						Sample inclusion tests	Pretests prior to intervention					
Case	G	Age Y/M	Fam.Risk	Lang.Cons.	Parents ed. level (years)	STAS pseudo word reading	WISC IV block design	WISC IV similarities	WISC IV digit span	WISC IV comprehension	WISC IV matrix reasoning	STAS pseudo word dictate
2	Boy	10/01	n.a.	No	12	−2.64	10	10	6	8	11	−3.2
3	Boy	10/00	Yes	Yes	12	−2.29	11	7	5	7	10	−3.7
4	Girl	13/02	No	No	15	−2.25	9	8	6	9	9	−0.8
6	Girl	14/00	Yes	Yes	15	−2.74	10	11	3	6	9	−2.7
7	Boy	11/01	Yes	No	15	−2.73	12	9	6	10	13	−2.2
8	Boy	11/08	Yes	Yes	15>	−2.52	13	11	4	8	7	−2.2
9	Boy	11/02	Yes	Yes	15>	−2.73	12	12	7	9	7	−1.2
10	Boy	12/03	Yes	Yes	15>	−2.52	19	12	6	12	12	−0.8
11	Boy	14/11	n.a.	Yes	15	−2.17	13	6	4	12	10	−2.1
12	Boy	13/06	Yes	Yes	15>	−3.40	11	9	2	10	9	−4.3
13	Boy	11/05	Yes	Yes	15>	−2.31	14	7	4	8	8	−1.4

*Note:*

G = gender

WISC IV: scaled scores

STAS: Norwegian standardised test for reading and spelling, standard deviations from the age mean score

Case characteristics were assessed by a questionnaire to the parents concerning the child’s early development, school history, socio economic factors and family risk factors. Lang.Cons = Parents’ concerns about early language development

Fam.Risk = family risk – dyslexia.

n.a. = not available

For all the participants, the reason for the referral to the Regional Department for Speech and Language Disorders was the need for new directions in reading instruction. Concerning individual dyslexic profiles, Cases 2, 3 and 12 were described in the school reports as ‘non-readers’. The remainder of the sample had slightly better reading skills, although not fluent readers. Rather than using decoding, most of the children recorded a high presence of different sorts of guessing strategies, typically displayed by decoding from random phonological cues instead of decoding each letter.

Most of the participants were students in public schools, but Cases 7 and 11 were enrolled in full time special needs education due to school refusal problems. Case 7 were separated from the class most of the day and received one-to-one instruction at his home school. Case 11 were placed in a small group of students with different sorts of learning disabilities, also receiving most of the instruction individually. Case 6 attended a private sports academy. Case 3 was enrolled in the child welfare service and was placed in a new foster care family during the intervention period.

The study followed the ethical guidelines of the national ethics committee.

### Intervention

**Fig. 2:**
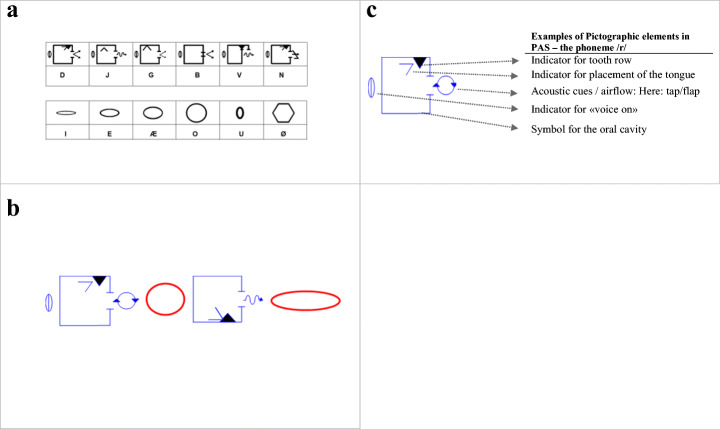
Pictographic articulatory system-PAS. **a**: Examples of how single phonemes are depicted in the PAS “alphabet”. **b**: PAS card ‘spelling’ of the word ROSE [2 ru:sə ], spelled in Norwegian. Cards contain blue script for consonants and red script for vowels. Consonant cards contain indicators for voice, placement of tongue and acoustic cues. Vowel cards symbolise the shape and the opening of the mouth when pronouncing a vowel. **c**: Illustration of the basic pictographic elements in PAS exemplified by the PAS symbol for the phoneme /r/.

The intervention programme aims to teach students a supplementary path to develop decoding and spelling skills by using pictographic symbols from the acoustic and articulatory features of each speech sound. The pictographic cards are based on singular vowels and consonants in the Norwegian alphabet. Consonant cards consist of indicators for voice, placement of tongue and acoustic cues. Vowel cards symbolise the shape and opening of the mouth when pronouncing a vowel. Figure 2 depicts how the word ‘ROSE’ is spelled in PAS.

**Fig. 3:**
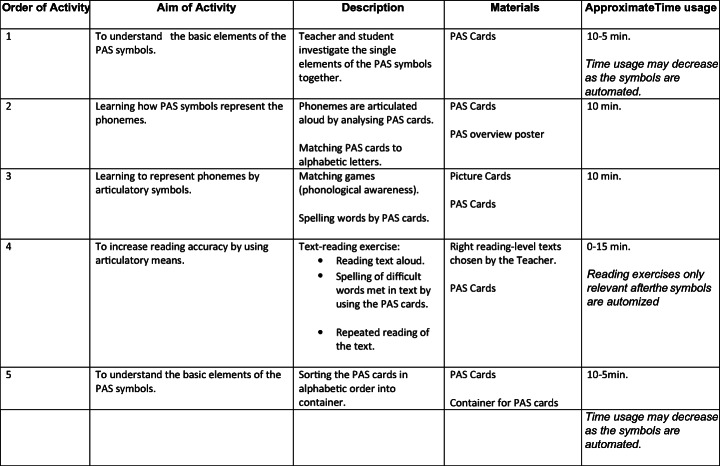
Articulatory consciousness training - learning activities involved in the intervention programme. Brief description of the content of the intervention programme, individual sessions, a`45 min. , four times a week for 8 weeks. The order and content of activities were the same for all sessions, but time usage may vary. For further information see Supplemental material

The intervention programme was constructed for the purpose of this study and consists of five learning activities. The intervention material consisted of PAS cards and a poster with the PAS symbols. Additional materials included mirrors, alphabet letter cards, pictures of objects and right reading level texts for the reading exercises. Details of the intervention programme are presented in Fig. 3. The intervention programme was introduced to the students as ‘a new way of reading’, and they were told that they were going to learn ‘secret signs’. Local, experienced teachers, the majority with additional training in special needs education, received individual supervision (mean of 1.5 h) in how to teach the programme. Further, individual supervision was also provided during the intervention period.

The teacher delivering the programme were for all participants the same as those who gave the children the special needs education prior to the intervention. The intervention programme did not presuppose the teachers to be trained experts in PAS. Instead, it was emphasized that the student and the teacher should explore the system together, and the teachers were encouraged to apply a dialogic approach and facilitate a collaborative atmosphere.

### Design and procedure

This study applied an adapted version of a non-concurrent multiple baseline/probe design across participants (Baer et al., 1968; Christ, 2007; Horner & Baer, 1978), with repeated testing over 18 weeks. More specifically, there were five pre-intervention measurement occasions, corresponding to a control condition, eight measurement occasions during the intervention and five post-intervention measurement occasions. For the baseline-phase, we used a probe design, with breaks of varying lengths between the measurement occasions. The reason for this was to avoid fatigue to the testing procedure and reduce the risk of practice effects (Horner & Baer, 1978). For the intervention phase, we used weekly measurements for all the occasions. This is because we desired detailed information concerning the students’ progress in learning to use the PAS symbols. In the post-phase, measurements were as in the baseline phase administered in ‘probes’. This was done to get more valid information about possible sustained effects from the intervention.

**Fig. 4:**
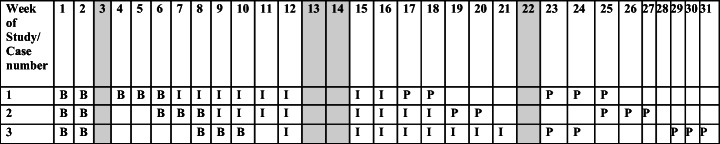
Individual patterns of weekly measurement occasions during three phases of study. Note: B = baseline, I= intervention, P= post. Empty fields: school holidays (marked) and scheduled stays (multiple probe design). The study includes three replications across three participants (*n* = 9) and four single replications using the pattern of the first participant

Cases 2–10 were separated in two groups, and the intervention onset was staggered across the participants in randomised order. Cases 11, 12 and 13 were recruited later in the process, introduced to the intervention at different time points and followed the structure of the first set of participants in the original design. For details concerning the structure of the measurement procedure, see Fig. 4.

At all the measurement points, the assessment followed the same procedure and took about 20 min. The testing was conducted by the first author or by professionals from the School Psychology Service.

The children received 32 hours of intervention distributed in four weekly sessions for eight weeks. Further, the teachers answered a short questionnaire about the organisation, content and extent of the special need’s education delivery in the periods before and after the intervention.

### Fidelity

All teachers filled in a log form for each lesson and briefly described how the intervention programme had worked out. All lessons were audio recorded. Ten percent of the recordings were rated, and the correspondence between the recordings and the log form were close to 100%. The measurement sessions were also audio recorded. A random selection of 15% of the sessions was picked out for each participant and rated to make sure that the procedure was followed. One student was removed from the sample due to procedural failures. There was 100% procedural fidelity for the remainder of the sample.

### Outcome measures

#### Weekly measures

Measurement tools for the baseline, intervention and post-phases were constructed for the purpose of this study. To minimise threats to internal validity from the testing effects, we constructed alternate forms for the reading and spelling tests for each testing point. All measures were constructed by reusing test-items that were randomly drawn from a test-item pool for each word category (Regular words, Pseudo words, Irregular words).

The reading measures, used twice at each measurement occasion, were made in 36 (2 × 18) unique versions and the spelling test in 18 unique versions.

#### Decoding

##### Regular words

We selected regular words from a database of the 500 most frequent Norwegian words (Norwegian word frequency list https://www.korrekturavdelingen.no/ord-uttrykk-frekvensordliste-500-vanligste-norsk.htm), which were separated in nine groups from their level of phonological complexity. The words in Groups 1 and 2 were simple c/v or v/c words with two graphemes, followed by Group 3 consisting of cvc words and then, successively, vcc, cvcv, cvcc, ccvccv, cccvccv and cccvccvc. For each word group, we made a pool of 22 words. The alternate forms of the reading tests were then constructed by randomly picking a selection of six words from each group, totalling 54 items. The scoring criteria were the number of correct words read in 1 min. A word was counted as correctly read if all the graphemes were articulated.

##### Pseudowords

The Pseudo Word Test was constructed to be similar to The Regular Word reading Test both in structure and level of phonological complexity (but with nonwords instead of words). For each word group in the ‘regular word pool’, we therefore constructed pseudowords with a similar phonological structure, creating 9 pseudoword groups. The Pseudoword Test, also containing 54 items, was thereafter constructed by following the same recycling procedure as for the regular word test. The scoring criteria were the number of correct words read in 1 min. Words read in a phonologically acceptable way were scored as correct if all the graphemes were articulated.

##### Irregular words

For the construction of the Irregular Word Test, we selected irregular words both from existing reading tests and from the word frequency list. The words were categorised based on the number of letters and syllables and separated in three groups. The first group (22 words in total) mainly consisted of high-frequency irregular words with two or three graphemes. The second group consisted of 44 one-syllable words, whilst the third pool consisted of 44 two-syllable words. As for the regular words and pseudowords, we made 36 versions, each containing 54 items. The scoring criteria were the number of words read in an orthographically correct manner in 1 min. Thus, a pronunciation that was phonologically correct but orthographically incorrect was scored as zero.

All word reading tests were administered twice, at each measurement time, with two alternate forms. The reliability of the reading tests was measured by correlating the scores from the two alternate forms at each time point. The average correlations across time points are for the regular words .968, for the pseudowords .898 and for the irregular words .883.

### Reading accuracy

Since ‘guessing strategies’ were highly frequent within the sample, we were interested in whether the intervention could improve the students’ reading accuracy. Therefore, we calculated the reading accuracy level to separate reading accuracy from reading speed (Juul et al., 2014). Accuracy was defined as the percentage of correctly read words out of the number of total items passed in 1 min.

#### Spelling

The construction of the alternate forms of the pseudoword spelling tests followed the same procedure as that of the reading measures, and the words were selected from the same pool as the pseudoword reading test. We picked four items for each level of difficulty, totalling 36 items in nine blocks. The time limit was 4 mn.

#### Transposition of pictographic symbols

The transposition test was also constructed using alternate forms (13 in total) and aimed to measure progress in the use of the articulation cards. Each test consisted of 24 pseudowords scripted in the font of the articulation cards. The pseudowords were selected from the same pools, as described above. They all had a vc, cv, vcc or cvcv structure and consisted of two to four symbols (letters). The scoring criteria were the number of pictographic words transposed into a correct alphabetic script in 4 min (max score was 24). Supplementary scoring also included the number of ‘pictographic graphemes’ transposed correctly into alphabetic graphemes (max score for this was 64).

#### Pegboard test (control task)

The pegboard test was considered unrelated both to the instruction provided by the intervention programme and to concurrent classroom teaching across subjects, and was mainly conducted to control for training effects from repeated testing. The materials used in this test were simply a pegboard and staples, and for each testing, the participants were asked to put as many staples as possible in a vertical line on the pegboard, with a time limitation of 30 s. For each measurement occasion, this was administered twice.

#### Standardised pre- and post-tests

To examine the effects of the intervention on measures not involved in the weekly measurement procedure, one word reading test (containing four subtests) and one pseudoword reading test (containing three subtests) from the STAS battery (Klinkenberg & Skaar, 2003) were administrated as pre- and post-tests. This battery is standardised and normed on a Norwegian sample from the second to tenth grade. The scoring criteria were the number of correct words read out loud in 40 s. Composite scoring options for words and pseudowords were also standardised and normed.

Pre-tests took place prior to the onset of the weekly baseline measurements and the post-tests were administered in the postintervention phase of the study.

#### Social validity

The teachers’ evaluation was measured by a post-study questionnaire. This consisted of 17 questions regarding the students’ motivation, general efforts during the sessions and the individual benefits of the training in respect of changes in the reading and spelling strategies. For each element, teachers marked their assessment from six options, ranging from very poor benefits to very great benefits.

#### Analysis

In line with the current guidelines for evaluation of SCED, we used a multi-methodological approach for the analysis, including both visual inspection of data and effects size statistics (Maggin & Odom, 2014; Tate et al., 2016).

**Fig. 5:**
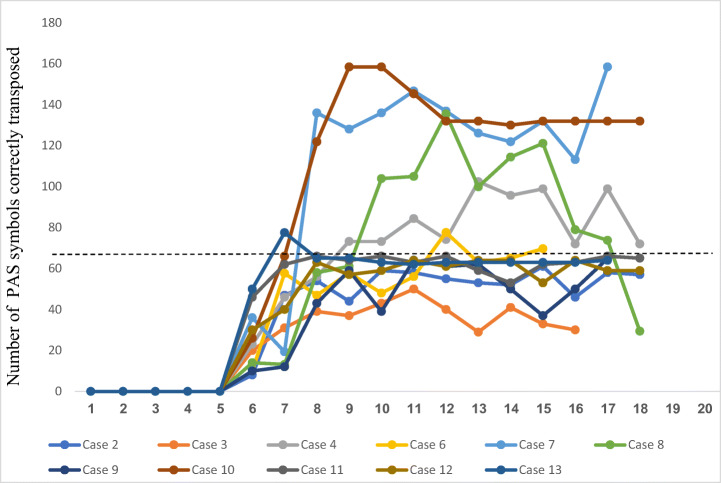
Results from the transposition test. Note: Pictographic symbols unknown to participants prior to intervention onset (week 6). Horizontal line: Ceiling level (64 symbols correctly transposed in 4 min). Scoring above the horizontal line = Extrapolated values (time bonus)

Further, the analyses were adapted to suit the current design. Since the effects on reading and spelling in this study are dependent on the transfer from learning the PAS symbols, we did not expect an immediate effect on the dependent variables after intervention onset (Klingbeil et al., 2017). However, the results from the transposition test showed that most students automatized the use of the PAS symbols during the first 2 weeks of the programme, which is depicted in Fig. 5. To calculate the effect from the reading and spelling outcomes, therefore, we only included data from week three of the intervention.

A mean score was calculated for the two forms of the reading (pseudowords, regular words and irregular words) and peg board measures administered at the same time point and plotted as new values for each measurement occasion. The intervention and post-test phase were merged in the analysis.

In the spelling and transposition tests, some students completed the task before the time limit, and they got a ‘time bonus’. The time bonus was calculated by estimating an extrapolated score based on the actual time spent and the ratio of speed to the number of correct responses obtained within the time limit of 4 min.

#### Visual inspection of data

Traditionally, intervention effects in single-case designs have been analysed through visual inspection of graphic charts of the repeated measurements. Visual inspections evaluate whether the intervention is followed by a change in the pattern of the data or not (Gast & Ledford, 2014). The inspection includes evaluation of (1) *Level,* which refers to whether the data points show a stable centring around the median value of the particular phase, and whether there are differences in median or mean values between the phases. (2) *Trend*, which refers to an inspection of the slope (gradient of the line) for the data series within each phase, to examine whether the trend direction is accelerating, decelerating or neutral and to what extent there is variability of data points around the trend line. (3) *Overlap*, which refers to the percentage of data points in the intervention phase that does not exceed the baseline median value (Gast & Spriggs, 2014; see also Lane & Gast, 2014).

**Fig. 6:**
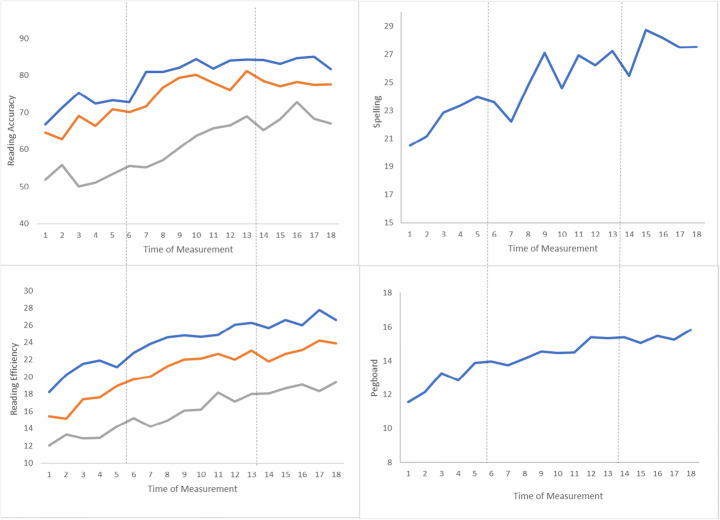
Full sample overview. Graphics of mean scores across dependent variables. Dotted lines represent phase changes. Left panels: blue line = regular words; orange line = pseudowords; grey line = irregular words

For the visual data inspection, we conducted a full sample overview (depicted in Fig. 6) and graphic charts for each participant (Supplementary material). The graphic charts give a detailed overview of the characteristics of the data, and the individual progress during the intervention. In this study the visual data inspection was used in combination with effect size statistic to evaluate the intervention effect.

#### Effect size statistic

Current guidelines for evaluation of single-case designs lack consensus on which effect size statistic to use, and multiple measures are recommended (Kratochwill et al., 2010, 2013; Tate et al., 2016; Lobo et al., 2017; Wolfe et al., 2019). Here we use two effect size statistics, representing two different approaches to evaluation of within case effects: The standardised mean difference (SMD) (Busk & Serlin, 1992; Olive & Smith, 2005) based on the *d-*statistic (Cohen, 1988) and the Tau-U (Parker et al., 2011a; Parker et al., 2011b) adapted for non-parametric statistic. Additionally, to get a measure of the overall effect across participants, we also calculated the between-case effect using between-case standardised mean difference (BC-SMD) estimates developed by Pustejovsky et al. (2014), see also Valentine et al. (2016).

The *SMD* expresses the effect in standard deviation units based on a comparison of the mean scores for each phase of the study and for each participant (Busk & Serlin, 1992; Olive & Smith, 2005). The effect size was calculated by using the online calculator provided at https://jepusto.shinyapps.io/SCD-effect-sizes. We used the pooled *SD* across all calculations. According to suggested standards (Harrington & Velicer, 2015), an estimate lower than 1 is a small effect, 1–2.5 a medium effect and over 2.5 a large effect.

The Tau-U combines non-overlapping techniques with control for baseline trends. The effect size estimate was calculated by using the online calculator available at http://www.singlecaseresearch.org/calculators (Vannest et al., 2011). The calculator uses a two-step procedure: In the first step, the baseline trends were evaluated. In the next step the percentage improvement from baseline to intervention were calculated by comparing all pairs of data from the two phases. The evaluation in both steps is based on Kendall Rank Correlations (Parker et al., 2011). When significant baseline trends were detected these were corrected in the calculation of effect size estimate. Based on suggested standards, a Tau-U estimate lower than .20 is a small effect, from .20–.60 a moderate effect, from .60–.80 a large effect and more than .80 a very large effect (Vannest & Ninci, 2015).

The BC-SMD was calculated by using the calculator available at https://jepusto.shinyapps.io/scdhlm/. The BC-SMD uses a two-level model with a within-case regression model at the first level and a between-case variation at the second level. The standard applies a design comparable effect size using the same benchmarks as those of Cohen’s (1988) *d*, i.e. small effect = 0.10, medium effect =. 30 and large effect = .50. The calculator requires a specification of both the fixed and random levels in the baseline phase and a fixed level in the intervention phase. Further, we specified the models by using the criteria suggested by Wolfe et al. (2019): If the treatment effect across the participants differed by more than 10% of the scale on the y axis, we added a specification for a random level in the intervention phase. To determine whether to include specifications for trends in the models, we first inspected the graphic charts (Wolfe et al., 2019). If all participants had a clear and visible trend, we specified a fixed trend in that phase. For specifications of random trends, we converted the criteria from Wolfe et al. and adapted them to our sample size. They specified random trends if one out of three participants displayed a clear trend. We converted this to one-third of the sample (33.3%) and specified a random trend if four out of eleven participants showed a visibly clear trend. For all models, we choose the restricted maximum likelihood (RML) estimation method.

## Results

### Visual inspection

#### Overlap

Overlap was evaluated by the Tau-U, which indicated only minor overlap in the data points between the two phases (Table 4).

#### Trend

The inspection of the within-phase trends was done for each participant by using the ‘freehand method’, i.e. visual inspection (Gast & Spriggs, 2014). Generally, the inspection of individual charts revealed minor, but still visible, changes in trend direction between the phases (for details see Table 2 and Supplementary material). Figure 6 shows a small, but noticeable change associated with the intervention onset for the full sample overview, most pronounced in reading accuracy and spelling.

**Table 2:** Descriptive results

Outcome measure									
Case number	Phase of study	Regular words		Pseudowords		Irregular words		Spelling	Peg board
		Median	Accuracy	Median	Accuracy	Median	Accuracy	Median	Median
2	Baseline	**10.5**_**d**_	46.19	**9**_**d**_	44.39	**9**_**d**_	46.15	10_n_	**10.0**_**n**_
	Intervention	**14**_**a**_	54.86	**11.5**_**a**_	50.94	**8**_**n**_	41.0	**17a**	**12.5**_**n**_
	Post	16_n_	62.45	**13.5**_**d**_	58.48	**13.5**_**a**_	53.50	**16**_**n**_	**13.5**_**n**_
3	Baseline	14_a_	75.99	**13**_**n**_	86.67	3.5_n_	27.64	**16**_**n**_	**13.5**_**a**_
	Intervention	**13.5**_**n**_	84.93	**13.5**_**n**_	88.333	4.56_n_	38.24	**16**_**n**_	16.0_a_
	Post	**18.0**_**a**_	85.05	**10**_**d**_	64.84	**9**_**a**_	59.0	**18**_**n**_	**18.5**_**n**_
4	Baseline	**25.5**_**a**_	75.15	**14.5**_**a**_	59.20	**11.5**_**a**_	55.66	**22**_**n**_	**8.0**_**n**_
	Intervention	**31**_**a**_	84.42	20_a_	75.06	18_n_	71.96	**28.5**_**a**_	**10.0**_**a**_
	Post	**32**_**a**_	90.53	**26.5**_**n**_	85.65	**22**_**n**_	79.68	**32**_**n**_	**11.0**_**n**_
6	Baseline	**30**_**d**_	82.43	**23**_**a**_	75.38	**23**_**n**_	65.22	**21**_**n**_	**15.0**_**a**_
	Intervention	**32.5**_**a**_	86.73	**30**_**a**_	87.70	**26**_**n**_	76.93	**26**_**a**_	**15.5**_**n**_
	Post	**35**_**a**_	90.25	**32**_**a**_	90.98	**30**_**a**_	84.27	**26**_**d**_	**16.5**_**n**_
7	Baseline	19_a_	78.87	**14**_**a**_	66.67	**17**_**a**_	68.48	**21**_**a**_	**11.5**_**a**_
	Intervention	26.0_a_	86.73	23_a_	88.79	**19**_**a**_	90.08	**26**_**n**_	**14.0**_**a**_
	Post	28a	94.08	**25**_**a**_	92.62	**23**_**a**_	95.99	**26**_**n**_	**15.0**_**a**_
8	Baseline	**18**_**a**_	84.52	**18.5**_**n**_	87.60	**9**_**a**_	56.06	**20**_**a**_	**14.0**_**a**_
	Intervention	**21**_**a**_	88.36	**20.5**_**a**_	87.98	**13**_**n**_	78.34	**27**_**a**_	**16.0**_**n**_
	Post	**22**_**d**_	88.69	**24.5**_**a**_	91.51	**14**_**n**_	78.95	**30**_**n**_	**14.0**_**n**_
9	Baseline	16_a_	68.37	**12**_**a**_	58.04	**12.5**_**n**_	64.52	**16**_**a**_	**12.5**_**n**_
	Intervention	**24**_**a**_	91.14	**17**_**a**_	77.89	**16**_**a**_	76.16	**25**_a_	**13.5**_**n**_
	Post	**27**_**a**_	93.33	**19**_**a**_	82.75	**18**_**a**_	81.10	**32**_**a**_	**15.0**_**n**_
10	Baseline	**19.5**_**n**_	75.22	**15**_**a**_	76.13	**12**_**d**_	57.19	**30**_**n**_	**17.0**_**a**_
	Intervention	**23**_**a**_	88.75	**25**_**a**_	89.85	**15**_**a**_	66.34	**32**_**n**_	**19.0**_**n**_
	Post	**25**_**n**_	92.64	**27**_**a**_	94.34	**13**_**d**_	62.97	**32**_**a**_	**22.0**_**a**_
11	Baseline	**29**_**n**_	92.81	**29**_**n**_	93.31	**27**_**a**_	89.08	22	14.0_n_
	Intervention	**32**_**a**_	96.57	**32**_**a**_	93.90	**28**_**n**_	92.89	25	**14.5**_**a**_
	Post	**34**_**d**_	96.24	**33**_**n**_	94.28	**27**_**n**_	93.72	27	**17.0**_**a**_
12	Baseline	**7**_**d**_	22.67	**1.5**_**d**_	8.7	2_d_	8.04	5_d_	**17.0**_**a**_
	Intervention	9.5_a_	42.30	6_a_	25.49	3_a_	16.15	**10**_**a**_	**19.0**_**n**_
	Post	**10.5**_**d**_	41.64	6_d_	20.47	4_d_	25.9	**11**_**n**_	**19.5**_**n**_
13	Baseline	**26**_**a**_	75.91	**21**_**a**_	76.04	**8.5**_**n**_	26.02	**29**_**a**_	**14.0**_**a**_
	Intervention	**32**_**a**_	81.38	**26**_**a**_	77.19	**14**_**a**_	36.74	**33**_**a**_	**15.0**_**a**_
	Post	**36**_**a**_	86.89	**28**_**d**_	82.47	**17**_**a**_	46.61	**34**_**n**_	**16.0**_**d**_

Note:

Median values: median number of correct read words in 1 min

Accuracy: percentage of correctly read words out of number of words read in 1 min

Median values in bold: stability criteria of 80% of the datapoints within the 25% interval of the phase median value

Lowered fonts: trend direction within phases, _a_ = accelerating trend, _n_ = neutral trend, _d_ = decelerating trend

To compare the within-phase trends in the data between the variables, we also calculated the within-phase improvement for all the dependent variables. This was done by calculating the change in the overall sample mean within each phase of study (i.e. the difference between the last measurement occasion in the previous phase.) The results show some within-phase improvement, ranging from 12 to17.2% (i.e. the percentage increase in scores from first to the last time point in the phase) in the baseline phase for all the dependent variables. Further, the total increase for the intervention and post phase of the study, were 29.2%, 33.3%, 42% and 32.8% for the regular words, pseudo words, irregular words and spelling, respectively.

The control test shows a different pattern of within-phase improvement, with most changes within the baseline phase and smaller increases in the intervention and post-phases of the study (21.9 % in the baseline phase and in total 11% from the last baseline measurement to the last post measurement). The results are displayed in Table 3.

**Table 3:** Within phase trends

	Within phase improvement			
	Baseline	Intervention	Post	Total
Dependent variable				
Regular words	12.0	30.9	−0,6	29.2
Pseudowords	15.9	30.7	3.86	33.3
Irregular words	14.1	31.5	7.83	42.1
Spelling	17.2	36.1	−2.21	32.8
Control	21.9	9.3	1.51	11.0

Note: Comparison of within phase improvements between the variables. Values are calculated as percentage increase from the first to the last measurement occasion (baseline phase) and from the last measurement occasion in the previous phase to the last measurement occasion in the current phase (intervention and post phase). Total: Percentage increase from the last measurement occasion in the baseline phase to the last measurement occasion in the post phase. All calculations are based on the mean raw scores for the full sample.

#### Level

Descriptive results from the weekly measures show improvements in all the dependent variables from the baseline phase to the intervention and post-phase for all participants. The average increase in the intervention phase was 37% for the regular words, 60% for the pseudowords, 45% for the irregular words, 40% for the spelling test and 23% for the control task. For individual differences, see Table 2.

To evaluate the variability of the data we utilised the Gast and Spriggs (2014) protocol. According to this, 80% of the data points should be within the 25% range of the phase median value. The visual inspection revealed that these were achieved for most variables and participants. The percentage of participants with stable data across all phases of the study was as follows: 54.5% for the regular words, 72.7% for irregular words and pseudowords, 63.6% for the spelling test and 81.8% for the control. For individual results, see Table 2.

### Intervention effects

#### Weekly measures

The Tau-U results showed that most students scored above the benchmark for ‘large change’, i.e. Tau-U = .60 on all the dependent variables (Vannest & Ninci, 2015). The number of participants with significant scores above the benchmark for large change was 91% for the regular words, 75% for pseudowords, 58% for the irregular words and 66% for the spelling test. The SMD effect sizes are in line with this. According to the suggested standards from Harrington and Velicer (2015), 91% of the participants had significant scores above the benchmark for medium effects on the regular word test. For the pseudo words, irregular words and spelling-test, these were 100%, 72.7% and 45.4%, respectively (see Table 3). The most consistent within-case effects in terms of the magnitude of the effect and level changes were seen on the pseudoword reading test and the standardised pseudoword composite (Table 6). The descriptive results for all the within-case effects are shown in Table 4.

**Table 4:** Within-case effects

Outcome measures											
		Regular words		Pseudowords		Irregular words		Spelling		Peg board	
Case number	Effect size measure	Estimate	*p* value/CI	Estimate	*p* value/CI	Estimate	*p* value/CI	Estimate	*p* value/CI	Estimate	*p* value/CI
2	SMD	**2.15**	0.47–3.82	**2.18**	0.90-3.46	0.08	−0.14–0.31	0.94	−12–2.01	**2.87**	1.45–4.29
	Tau-U	**0.67**	0.04	**0.89**^**#**^	0.01	0.30^#^	0.33	0.49	0.13	**0.93**	0.00
3	SMD	0.50	−0.35–1.35	0.01	−0.19–0.22	**0.66**	0.11–1.20	0.54	−0.52–1.60	**1.89**	0.65–314
	Tau-U	0.36	0.29	0.15	0.64	**0.69** ^**#**^	0.03	0.24	0.46	**0.84**	0.01
4	SMD	**1.84**	0.57–3.12	**2.33**	0.85–3.81	**1.46**	0.14–2.77	**2.37**	0.88–3.86	**2.23**	0.77–3.69
	Tau-U	**0.97**	0.01	**0.91**	0.02	**0.81**	0.03	**0.85**	0.03	**0.87**	0.02
6	SMD	**1.65**	0.31–2.98	**3.44**	1.75–5.14	**1.62**	0.40–2.83	0.14	−0.02–31	**1.26**	0.11–2.41
	Tau-U	**0.83**	0.01	**1.0**	0.00	**0.80**	0.01	0.58	0.09	0.45^#^	0.18
7	SMD	**1.39**	0.28–2.50	**2.10**	0.86–3.34	**1.81**	0.62–2.99	**1.43**	0.31–2.55	**2.48**	1.13–3.8 3
	Tau-U	**0.76**	0.02	**0.80**	0.01	0.58^#^	0.07	**0.63**	0.05	**0.92**	0.00
8	SMD	**1.32**	0.22–2.43	**1.09**	0.02–2.15	**2.38**	1.08–3.68	**1.63**	0.48–2.78	0.50	−0.52–1.53
	Tau-U	**0.67**	0.04	**0.67**^**#**^	0.04	**0.91**	0.00	**0.78**	0.01	0.28	0.39
9	SMD	**3.23**	1.81–4.65	**2.72**	1.41–4.03	**1.77**	0.64–2.90	**1.81**	0.68–2.95	**1.07**	0.03–2.10
	Tau-U	**0.83**^**#**^	0.00	**0.99**^**#**^	0.00	**0.77**	0.01	**0.83**	0.00	0.59	0.06
10	SMD	**1.89**	0.69–3.09	**4.27**	2.55–5.98	**1.81**	0.66–2.96	-0.43	−1.42–0.57	**1.90**	0.70–3.10
	Tau-U	**0.95**	0.00	**1.00**^**#**^	0.00	**0.77**	0.01	**0.83**	0.00	**0.83**	0.00
11	SMD	**1.89**	0.69–3.09	**1.87**	0.68–3.07	**0.15**	0.08–0.22	-0.28	−1.28–0.72	**1.70**	0.54–2.87
	Tau-U	**0.87**	0.00	**0.85**	0.01	**0.95**	0.00	**0.75**	0.02	0.45	0.15
12	SMD	**1.50**	0.37–2.62	**1.91**	0.71–3.12	**1.30**	0.21–2.40	**2.45**	1.13–3.77	**1.68**	0.52–2.84
	Tau-U	0.80	0.01	**0.78**	0.01	**0.78**	0.01	**0.78**	0.01	**0.78**	0.01
13	SMD	**2.87**	1.45–4.29	**1.83**	0.64–3.01	**2.01**	0.79–3.23	0.90	−015–1.95	**1.71**	0.54–2. 88
	Tau-U	**0.98**	0.00	**0.84**	0.01	**0.93**	0.00	0.47	0.14	**0.83**	0.00

Note:

SMD = standardised mean difference

# = adjusted Tau-U estimates (corrected for significant baseline trends)

Significant results, *p* value < 0.05 in bold.

Regarding reading accuracy, all students showed improvement in the rate of correctly read words from the mean level in the baseline phase to the mean level in the intervention/post-phase. The mean level improvement across the reading measures from the baseline to the post-test was 10% for regular words and pseudowords and 12% for irregular words. For individual results, see Table 2 and individual charts attached in the Supplementary material.

#### Between-case effects

The results from the between-case analysis show a significant positive effect on all the outcome variables, with effect sizes ranging between *d* = 0.32 to *d* = 0.67. This means a moderate to large effect according to the suggested standards for *d*-statistic (Cohen, 1988). In the analysis, we followed the procedure described above. Details are shown in Table 5.

**Table 5:** Between case effects

Outcome measure	BC-SMD	S.E.	CI	Baseline model	Intervention model
Regular words	0.53**	0.13	0.23–0.84	Level: fixed + random	Level: fixed
Pseudowords	0.67**	0.20	0.22–0.94	Level: fixed + random	Level: fixed + random
Irregular words	0.48**	0.15	0.13–0.83	Level: fixed + random, Trend: random	Level: fixed + random
Spelling	0.63**	0.14	0.31–0.94	Level: fixed + random	Level: fixed
Control	0.32**	0.11	0.06–0.57	Level fixed + random, Trend: random	Level: fixed

Note:

BC-SMD = between case-standard mean difference

SE = standard error

CI = confidence interval

**= *p* < 0.05

### Social validity questionnaire

The teachers provided positive evaluations of the students’ benefit from the training. In the post-intervention questionnaire, the teachers evaluated the students’ general benefit from the training on a scale from 1 to 6. All the participants scored in the 4–5 range. On a question about changes in reading and spelling strategies, the scores were in the same range. The teachers also reported the students’ effort in the sessions during the intervention, and for 10 out of 11 students, this was categorised as ‘better’ or ‘much better’ compared to the teachers’ experiences from prior special education sessions.

### Standardised pre- and post-tests

The results from the STAS test, administered prior to and in the post-intervention phase, show that on the pseudoword composite, most students had improved equivalent to approximately 0.5 *SD* when converting the raw score changes from pre- to post-test to the age norms for each participant. For the word-reading measures (including both regular words and irregular words), the improvement was between 0.01and 0.45 *SD*. See Table 6 for details.

**Table 6:** STAS pre- and post-tests

Case number	2	3	4	6	7	8	9	10	11	12	13
Mean scores											
STAS - word reading composite											
Pre-test	37	22	70	73	54	28	43	36	60	11	66
Post-test	43		90	88	70	53	64	49	82	12	84
Difference (*SD*)	6 (0.10)	n.a.	20 (0.35)	15 (0.23)	16 (0.29)	25 (0.45)	21 (0.38)	13 (0.22)	22 (0.34)	1 (0.01)	18 (0.32)
STAS - pseudo word reading Composite											
Pre-test	9	16	36	35	26	20	21	26	45	6	29
Post-test	24	33	37	42	39	40	35	36	59	1	43
Difference (*SD*)	15 (0.55)	17 (0.62)	18 (0.05)	7 (0.23)	13 (0.48)	20 (0.74)	14 (0.51)	10 (0.37)	14 (0.46)	-5 (-0.17)	14 (0.51)

Note: All scores are raw scores

*SD*: improvement expressed in standard deviation units from the age mean standard

n.a.= not available

### Control test

The pegboard test was conducted as a control for the practice effects due to repeated measurements. According to the Tau-U, 54.5% of the sample had significant scores above the benchmark for ‘large effects’ (Vannest & Ninci, 2015).

For the SMD, the percentage of participants with significant scores above the benchmark for the ‘medium effect’ was 91 (Harrington & Velicer, 2015) (Table 4). The BC-SMD estimate was significant with effect size *d* = 0.32 (Table 5).

## Discussion

This study evaluated the effects of an 8-week intervention aimed at improving phonological reading and spelling strategies in 11 students with severe dyslexia. The study results include both the students’ progress in automatizing the pictographic symbols and the possible transfer effects to alphabetic reading and spelling. In addition, we used a task unrelated to the intervention to control for testing effects.

The between-case results showed significant improvement on all outcome variables, with the most substantial effects for pseudoword reading and spelling and reading of regular words and slightly weaker effect on the irregular word measure. Since the training was primarily aimed at improving phonological strategies in reading and spelling, it is not surprising that the effect on irregular words, which required other reading strategies, was in a lower range.

The within-case effect size statistics showed significant improvement across the dependent variables for most participants. Although the effect size statistics showed some divergent results concerning the magnitude of the effects, there were consistent results for all the effect size statistics when it comes to whether there was an effect or not. This is in line with previous studies applying multiple effects size statistics in SCED (Olive & Smith, 2005; Wolfe et al., 2019). An exception was the spelling test, where the SMD measure indicated a more conservative judgement than the Tau-U measure for two participants, as well as the control test where the Tau-U seemed to be more conservative than the SMD for three participants.

Further, the results showed that most students automatized the PAS symbols during the first 2 weeks of intervention. The teachers also reported that the students were motivated to learn the ‘secret signs’ and generally put more effort in the training than in previous reading lessons. This suggests that the ‘articulatory way’ had some sort of appeal to this sample of students with severe reading disabilities.

## Interpretation of the findings

Repeated measures of the same variables are vulnerable to practice effects, which are a possible threat to the internal validity of single case studies (Gast, 2014). For this reason, we added a non-equivalent dependent variable (Shadish et al., 2002), the pegboard test, as a control. An important issue is that we not only found effects on the outcomes targeted in the intervention but also significant and small-to-moderate related effects. However, according to the descriptive results and the visual inspection, the average level of improvement on the pegboard test was nearly half the improvement on the reading and spelling measures, respectively, 23% for pegboard and 36–60% for reading and spelling.

Moreover, the evaluation of the within-phase trends showed a different pattern of improvement for the control test compared to the other measure. Most of the improvement was in the baseline phase, but for the other dependent variables, the improvement was recorded in the intervention and post-test phases. This suggests that the five measurement occasions during the baseline were sufficient to capture the practice effects from repeated testing.

It should also be noted that we used alternate forms for all the reading and spelling tests in order to control for practice effects, which was not done for the pegboard task. A study on testing effects in neuropsychological measures showed that the use of alternate forms of tests to some extent prevents testing effects on some tests, even though continued learning occurred when an advantageous test-taking strategy could be identified (Beglinger et al., 2005). However, for verbal memory measures, it has been demonstrated that alternate forms can eliminate practice effects (Roediger & Karpicke, 2006). Another study also indicated a general appearance of more substantial testing effects on motoric measures than on word reading (Levine et al., 2004).

Thus, it seems that the nature of the test plays a role in the magnitude of the testing effects, including when using alternate forms. The results from the pegboard test limit the conclusions that can be drawn from the study concerning the benefits on the primary outcomes. However, based on the line of arguments above, it appears that testing effects are not a likely explanation for all changes in the current study.

The results from the immediate and delayed post-tests show that, for most participants, the effect from the intervention remained after the 8 weeks of training. According to the post-study questionnaire, most participants continued to use elements from the intervention programme after completing the intervention. The increased effects at post-test can, therefore, be partly explained as a continuation effect. This indicates that less intensive training was enough to maintain the achieved effect; however, with the effects from the pegboard task as a caution, this could also be interpreted as continued testing effects. Nevertheless, the within-phase analysis showed minor improvement in the intervention and post-phase for the control test compared to the other dependent variables. Therefore, the continued effect is less likely to be due to the repeated measurements.

The pre-post assessments with the STAS battery indicate that the improvement, shown by the results from the weekly measurements, were also present at this standardised task. However, some reservations should be considered regarding this: Since the measurement procedure for the STAS subtests and the weekly measurements are quite similar (40 s reading aloud for STAS and 1 min aloud reading for the weekly measures), there might have been some practice effects. Also, since the recruitment of participants were made from extreme values at the STAS pseudo word sub tests, a possible effect of regression to the mean, i.e. the tendency for extreme values to move towards the mean when repeating the assessment, may also be an alternative explanation for this measure.

Although most participants took part in the general instruction in their respective classrooms during intervention, the literacy instruction in the classrooms were not adapted to the poor reading level of these marginalised students, i.e. the instruction did not include basic decoding and spelling exercises. This makes it less likely that the concurrent literacy instruction can explain the improvement in basic decoding and spelling skills. Further, because the intervention programme occupied all available earmarked special needs resources for the participants during the 8 weeks of the intervention, participants did not receive any other basic reading and spelling instruction than the instruction provided by the intervention programme during this period.

Notably, two students (Cases 2 and 3) scored below the benchmarks for the small effects on more than one of the independent variables and across two or more effect size statistics. For Case 3, poor results can probably be explained by personal situation changes during the intervention period. This case also showed weaker progress than the rest of the sample on the transposition test (Fig. 5), which means that the probability of transfer effects to reading and writing were less likely. The poor results for Case 2 seemed random, but a possible explanation may be stress, due to the time limitations of the tasks. This because his teacher report sustained positive changes in his reading and spelling strategies in natural settings, i.e. reading and spelling tasks without time limitations (social validity questionnaire).

In conclusion, this study exemplifies that articulatory consciousness training may have positive effects on reading and spelling outcomes for students with severe dyslexia. This is in line with previous studies that have found that students with most severe reading problems benefit most from articulatory consciousness training (Fälth et al., 2017; Trainin et al., 2014). Regarding the functionality of the current intervention programme, this study shows that most participants manage to use the articulatory symbols effectively after 2 weeks of intensive training. In a pedagogical perspective, this indicates that this method may serve as an additional instructional tool to clarify the phonological structure of scripted words for students with poor phonological skills. As shown, the results also indicate that the training had some sustained effect on the reading and spelling outcome for most participants, suggesting that the training may function as a bridge to alphabetic reading and spelling for those with most severe phonological deficiencies.

However, the results must be interpreted with caution as significant effect sizes were recorded for the irrelevant task. Furthermore, from the perspective of generalisation, since this study only included 11 participants, future studies need to focus on group comparisons with randomisation to determine results.

## Supplementary Information

ESM 1 (DOCX 1112 kb)
