# Supplementary material for: The impact of articulatory consciousness training on reading and spelling literacy in students with severe dyslexia: an experimental single case study
Source: Ann Dyslexia. 2021 Apr 30;71(3):373–98. doi: 10.1007/s11881-021-00225-1 (PMC8458204; doi:10.1007/s11881-021-00225-1)
Supplement: Supplementary file 1 — (DOCX 1112 kb) [file 11881_2021_225_MOESM1_ESM.docx]

**Supplemental material**

**The Impact of Articulatory Consciousness Training on Reading and Spelling Literacy in Students with Severe Dyslexia: An Experimental Single Case Study.**

***Annals of Dyslexia***

Anne Cathrine Thurmann-Moe

University of Oslo

Department of special needs education

and Statped (National service for special needs education)

Anne-Cathrine.Thurmann-Moe@statped.no

Monica Melby-Lervåg

University of Oslo

Department of special needs education

monica.melby-lervag@isp.uio.no

Arne Lervåg (corresponding author)

University of Oslo

Department of Education

a.o.lervag@iped.uio.no

1. **Graphic charts for each participant.**
   1. **Raw scores at each timepoint of measurement.**

**Y- axis: Raw scores, X-axis: Time of measurement. Vertical lines: Phase changes (Baseline, Intervention, Post). Note: Individual breaks from probe sessions and scheduled school holidays are not displayed.**


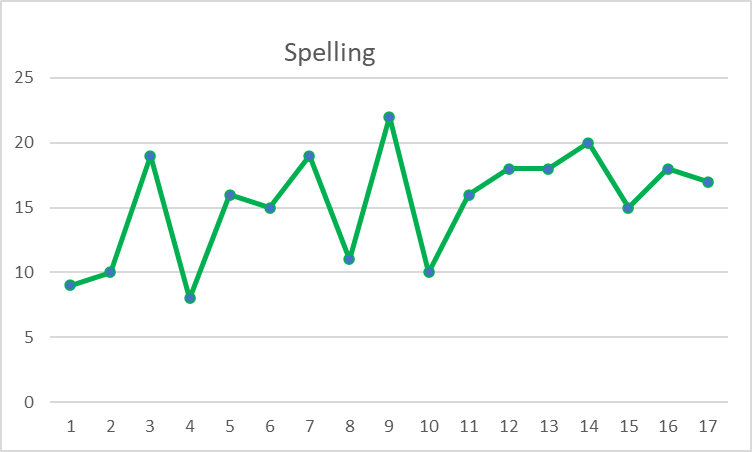

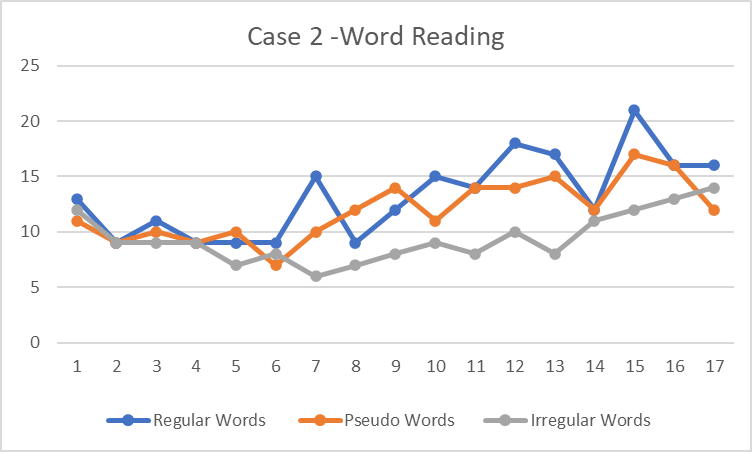

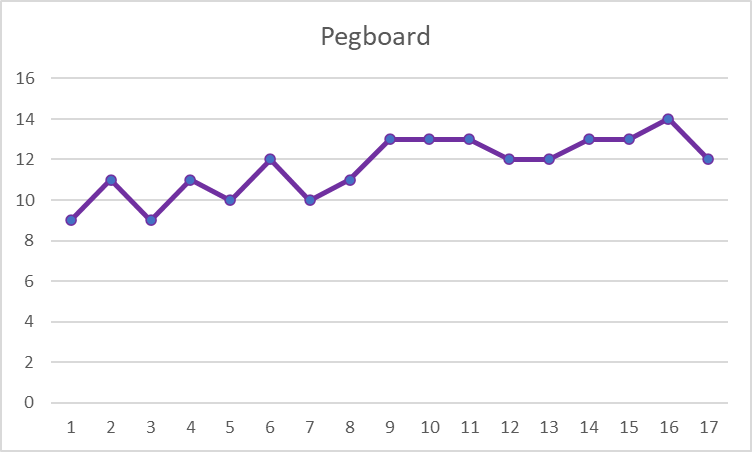

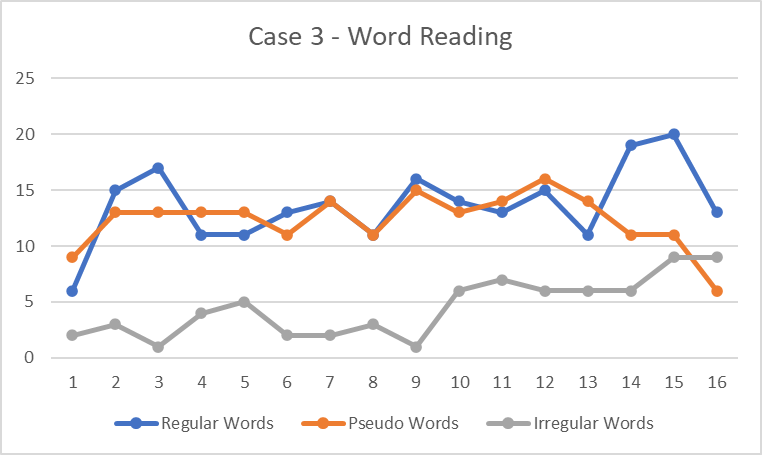

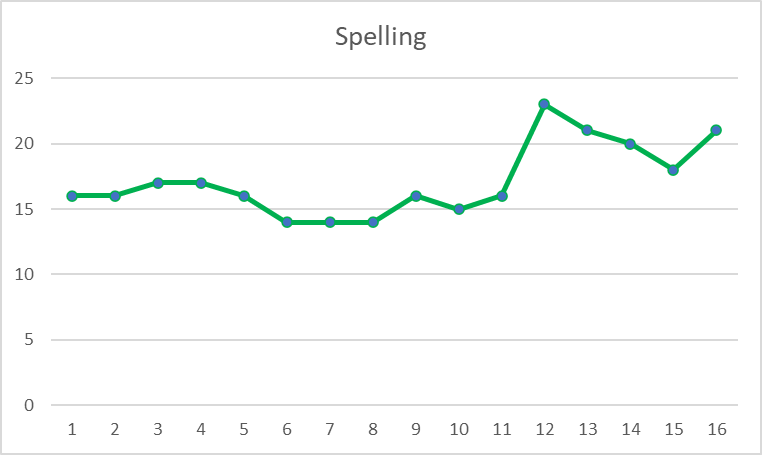

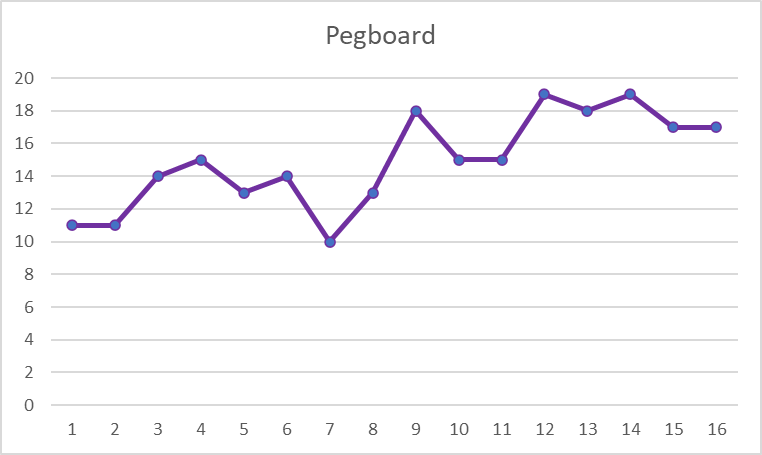

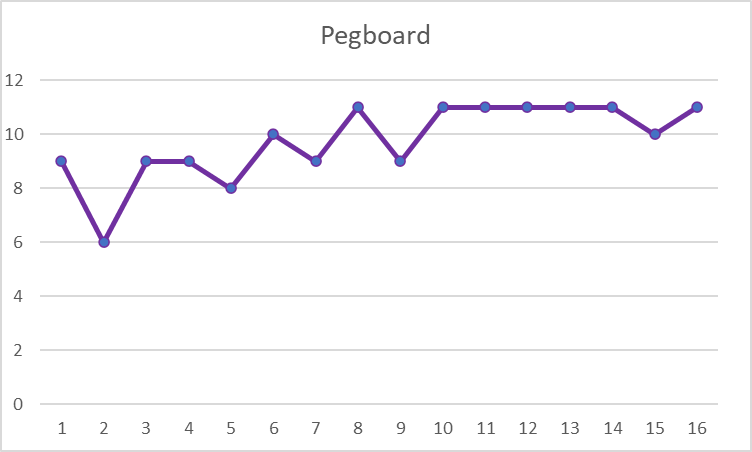

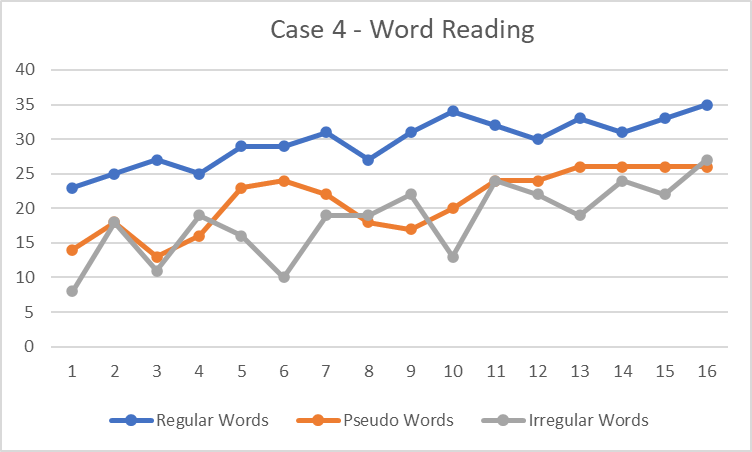

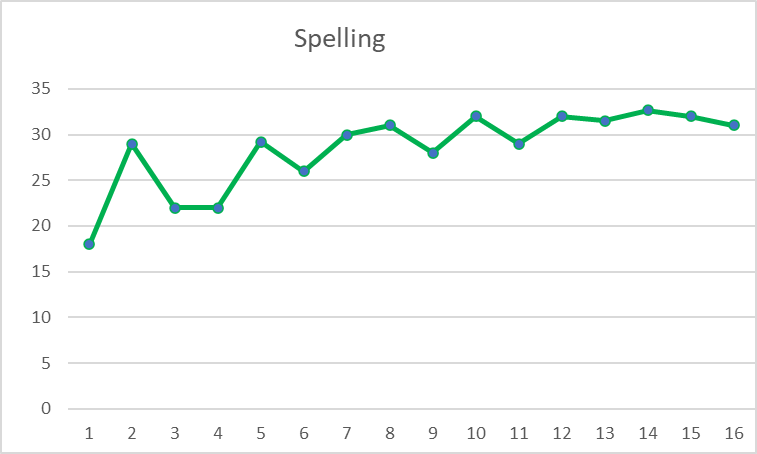

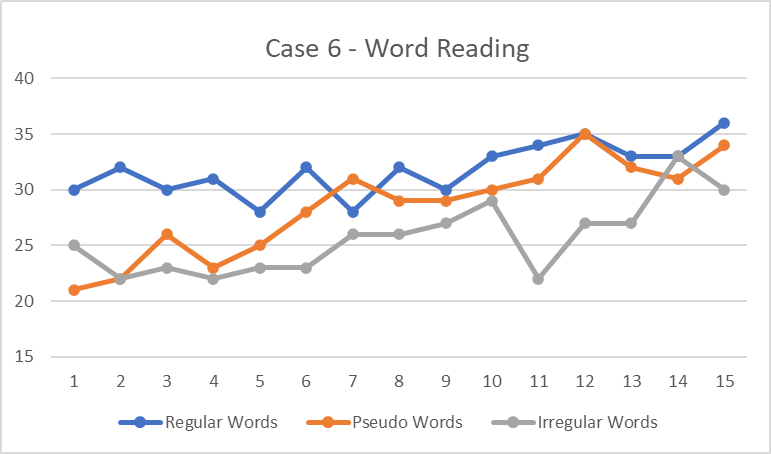

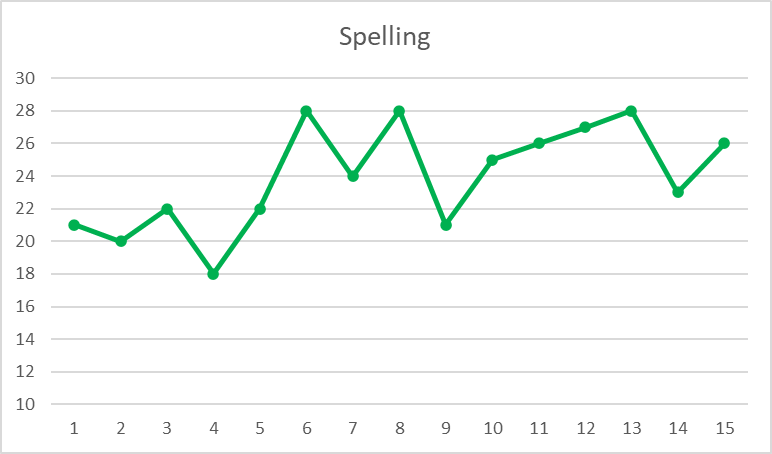

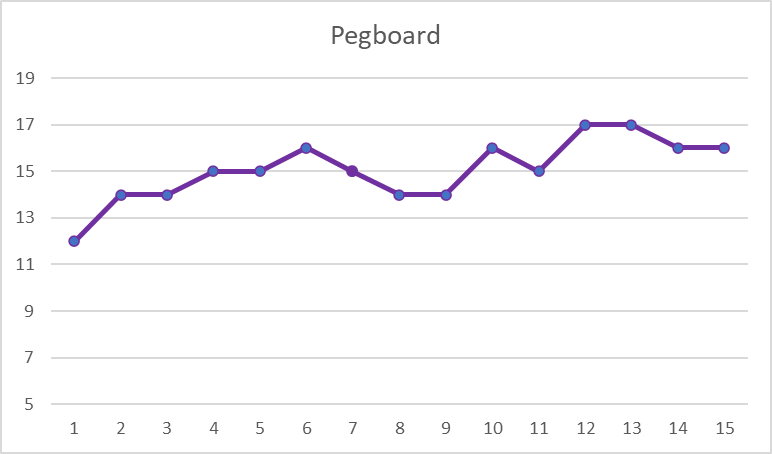

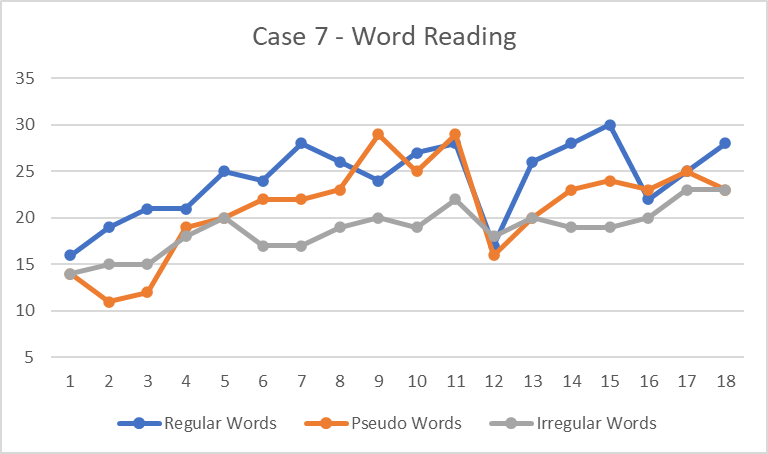

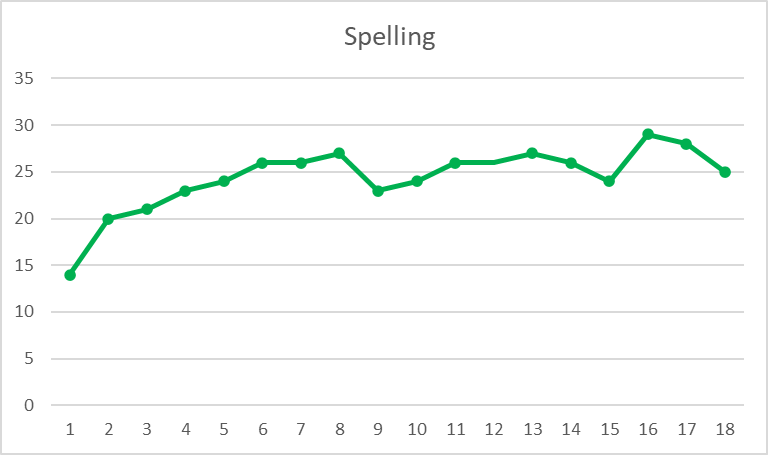

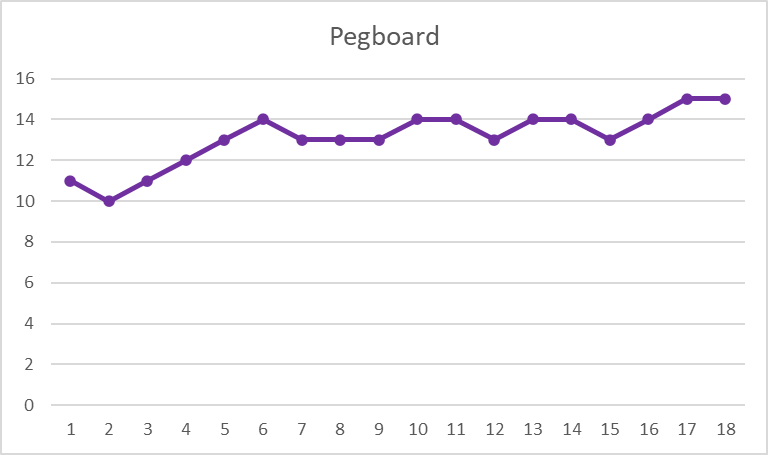

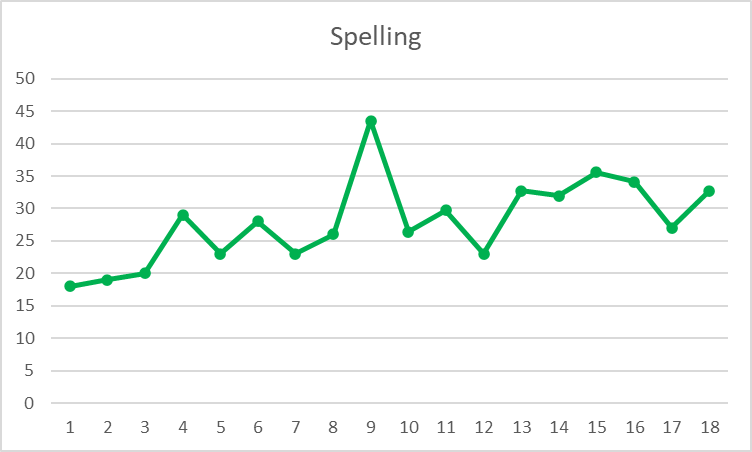

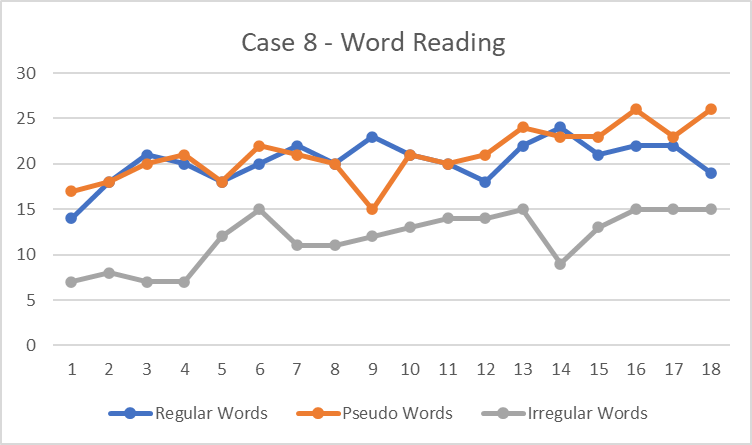

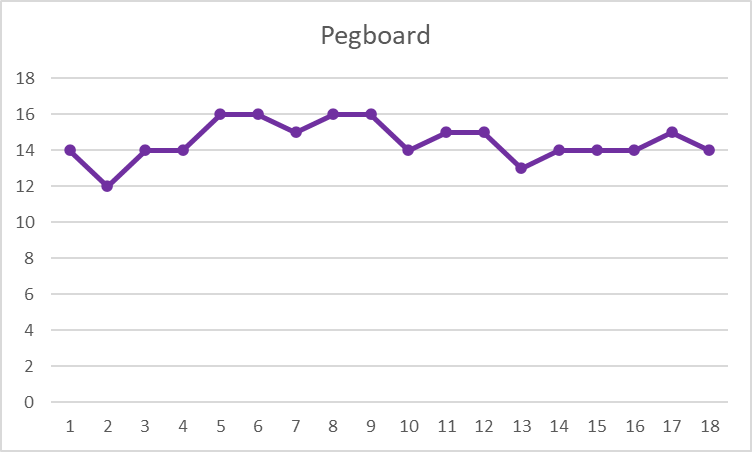

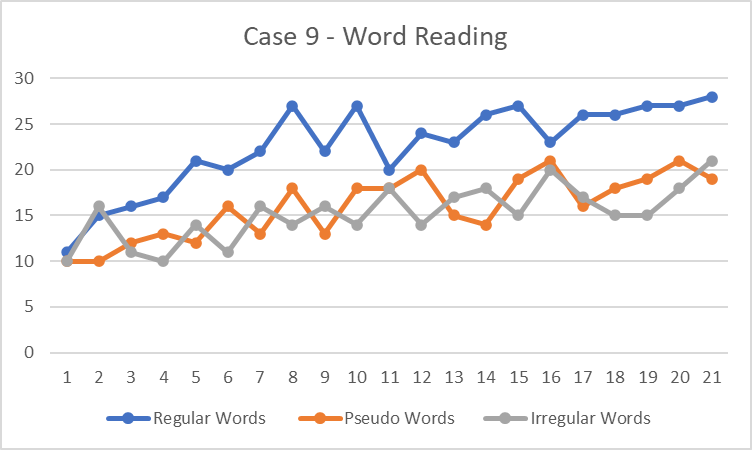

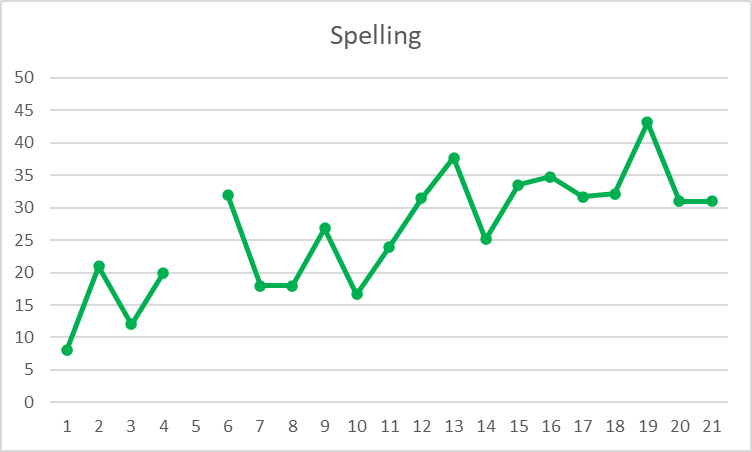

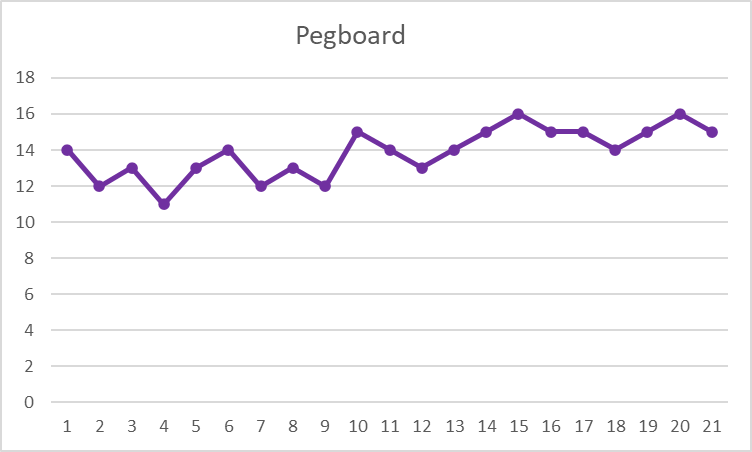

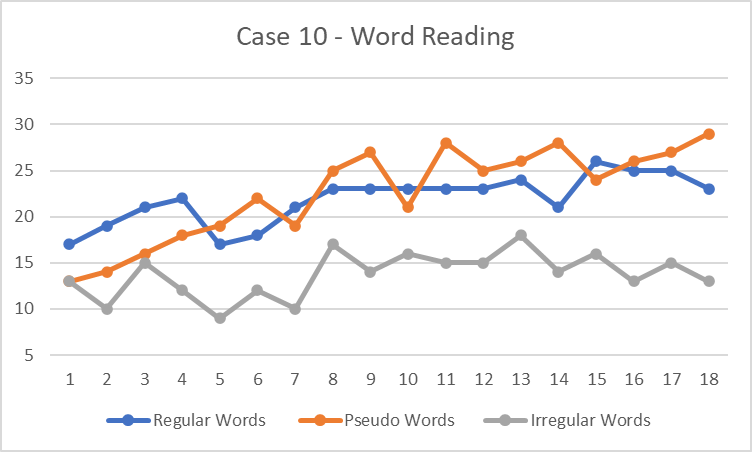

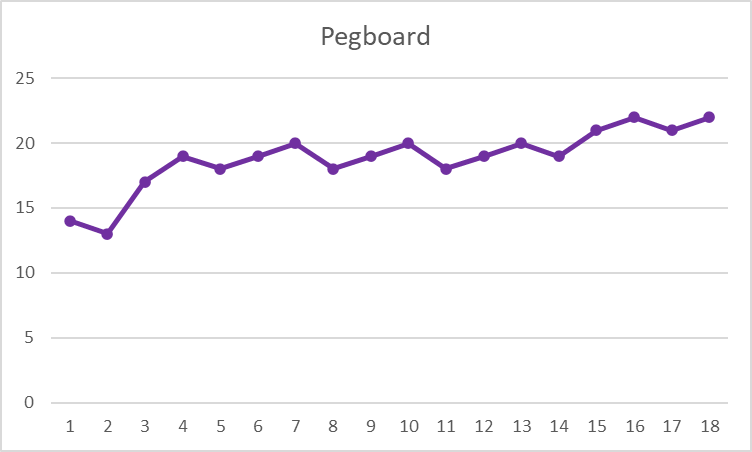

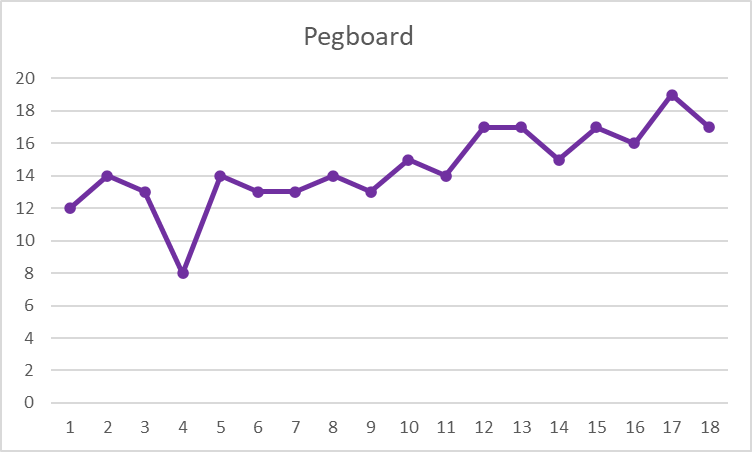

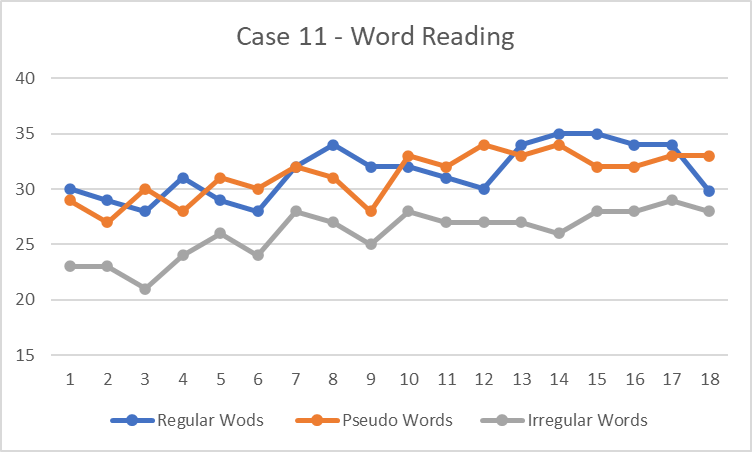

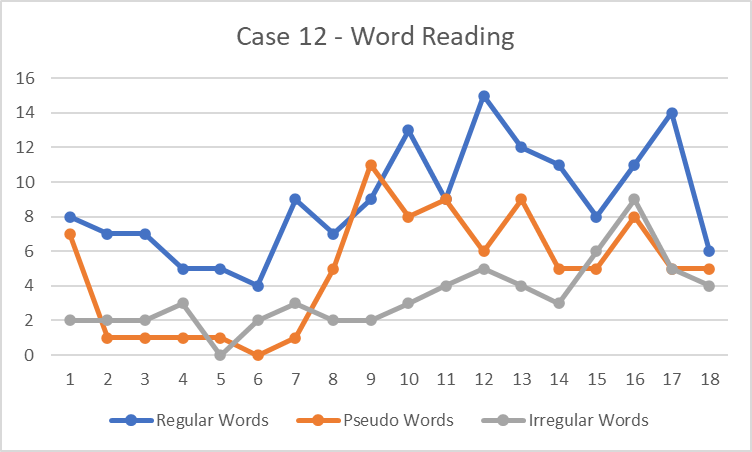

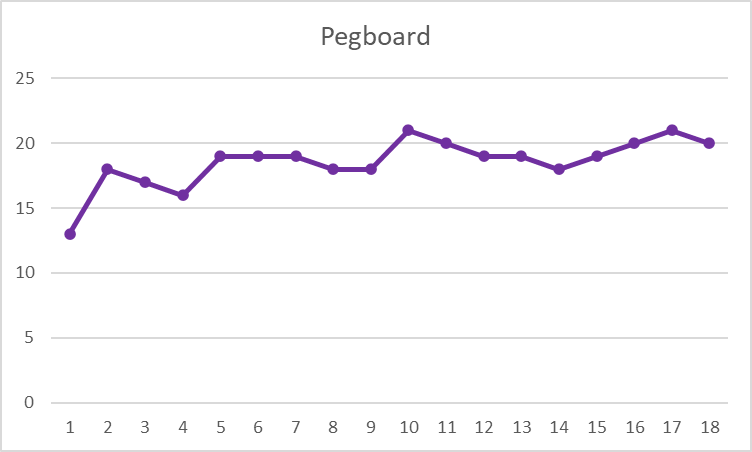

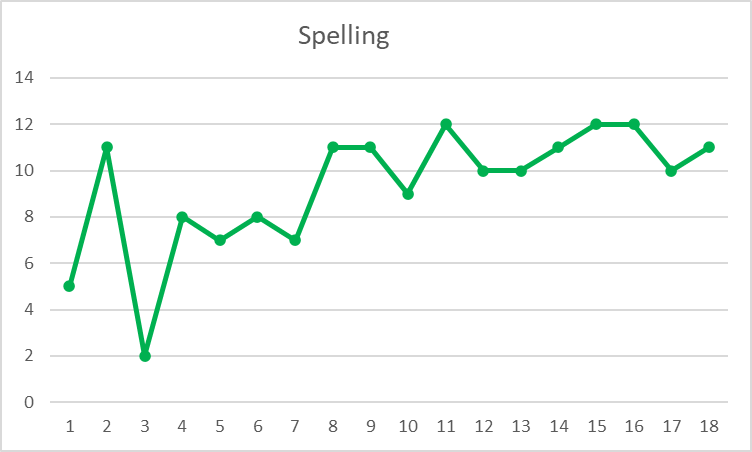

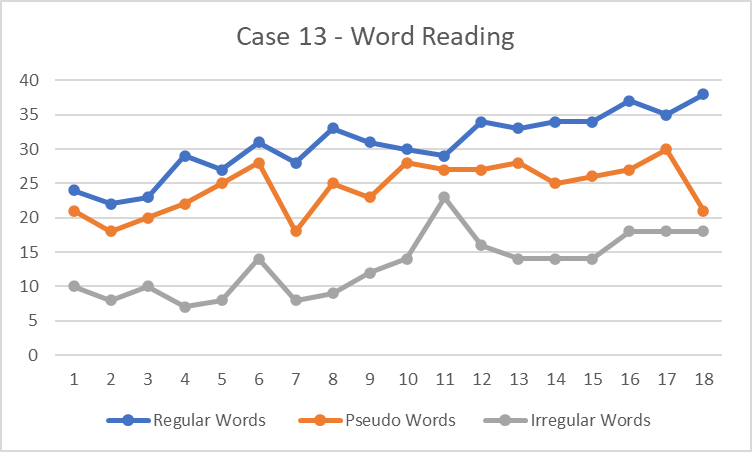

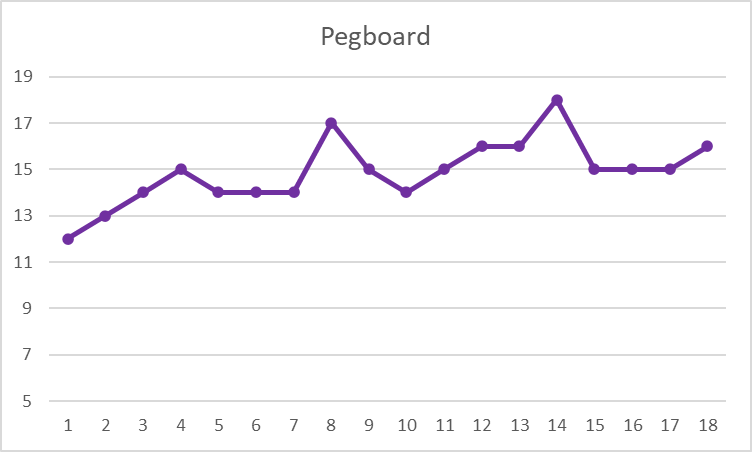


- 1. **Bar charts showing the level change of reading accuracy across three phases of study.**


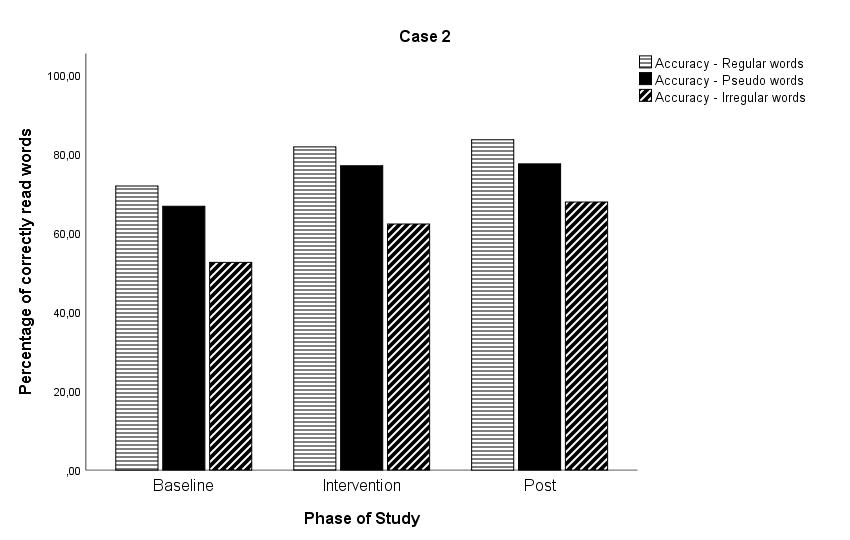


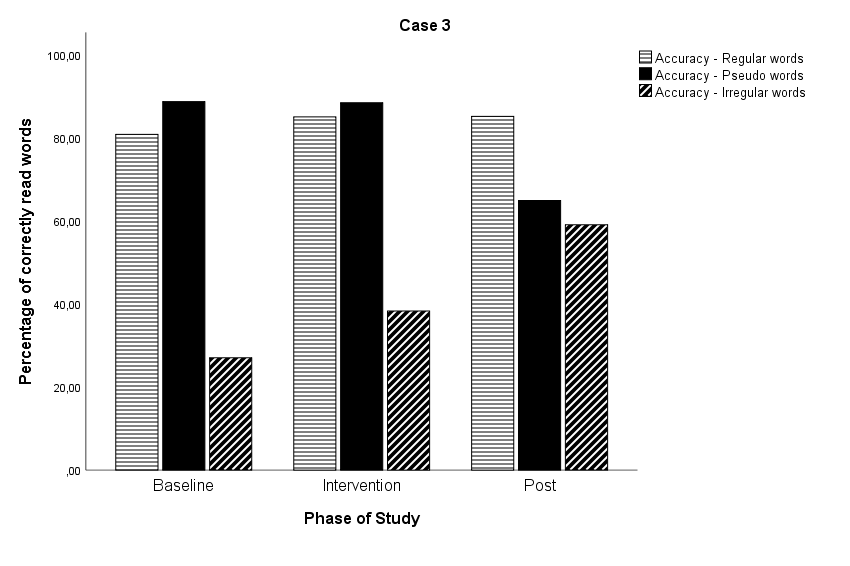


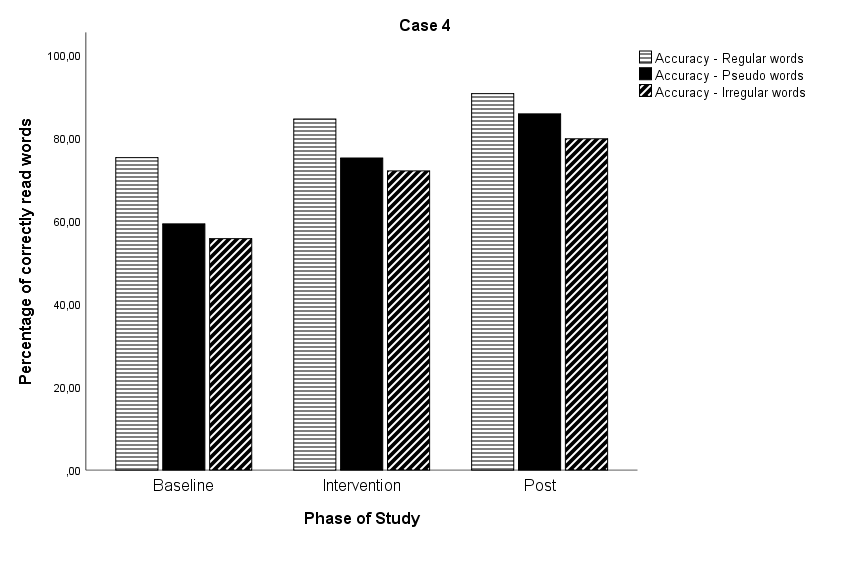


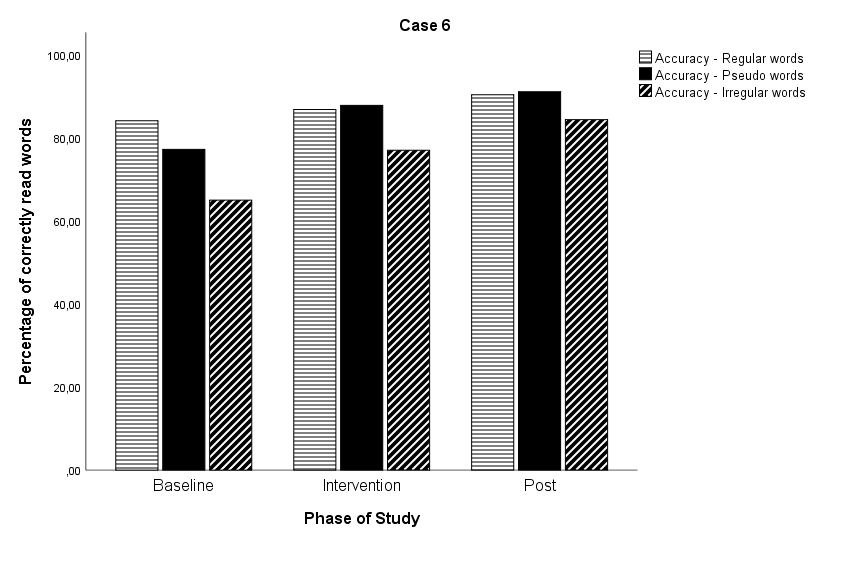


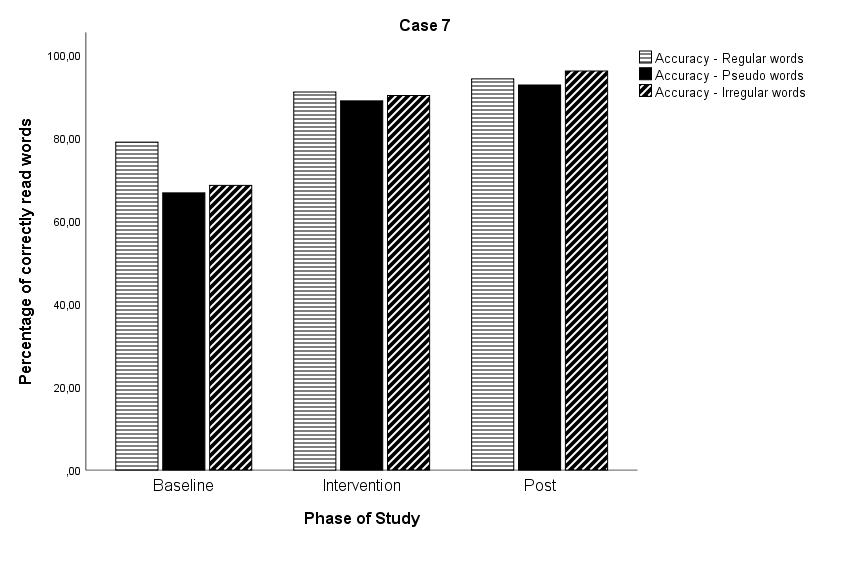


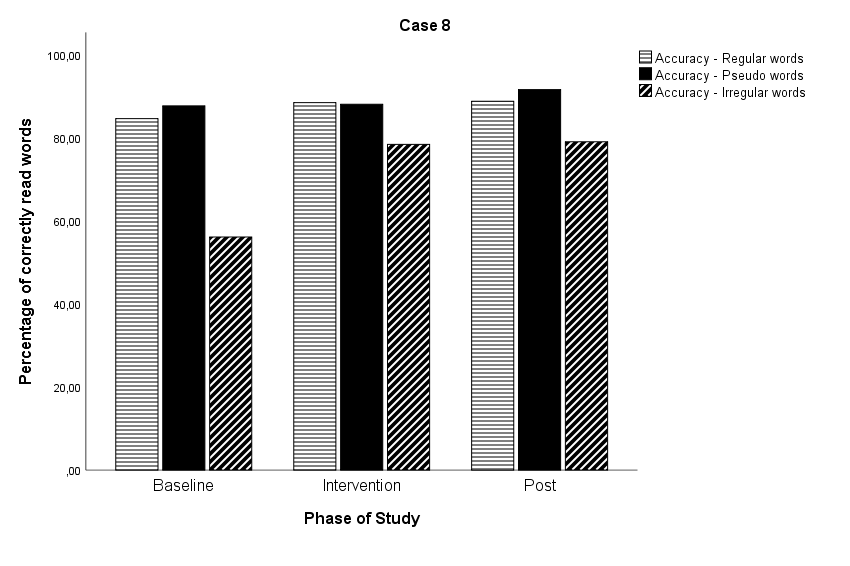


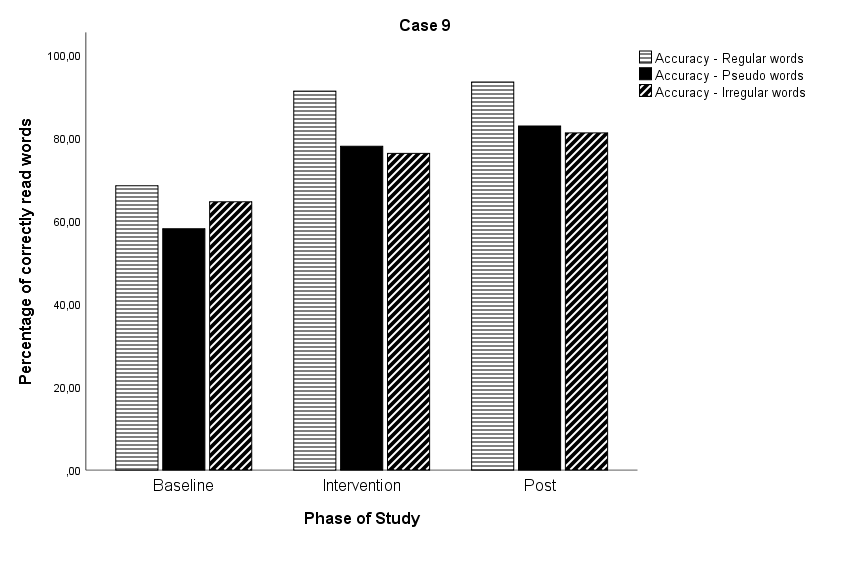


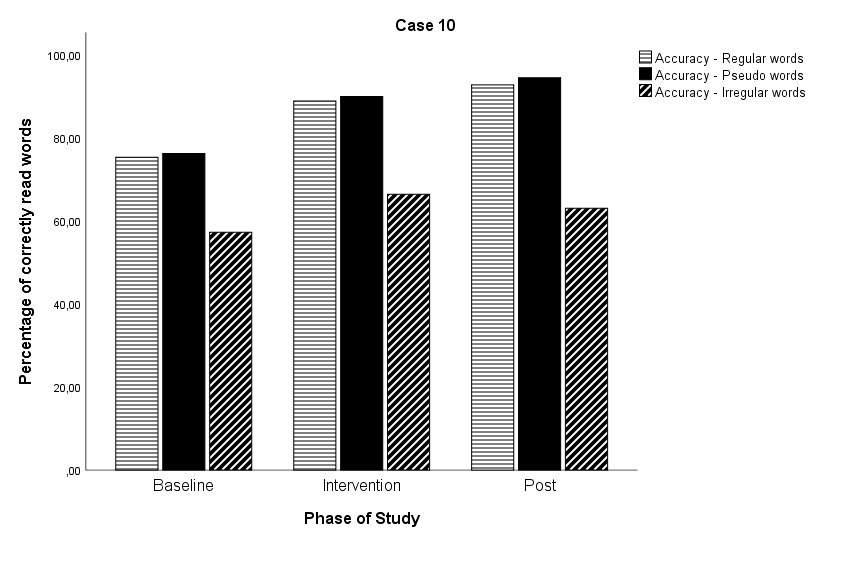


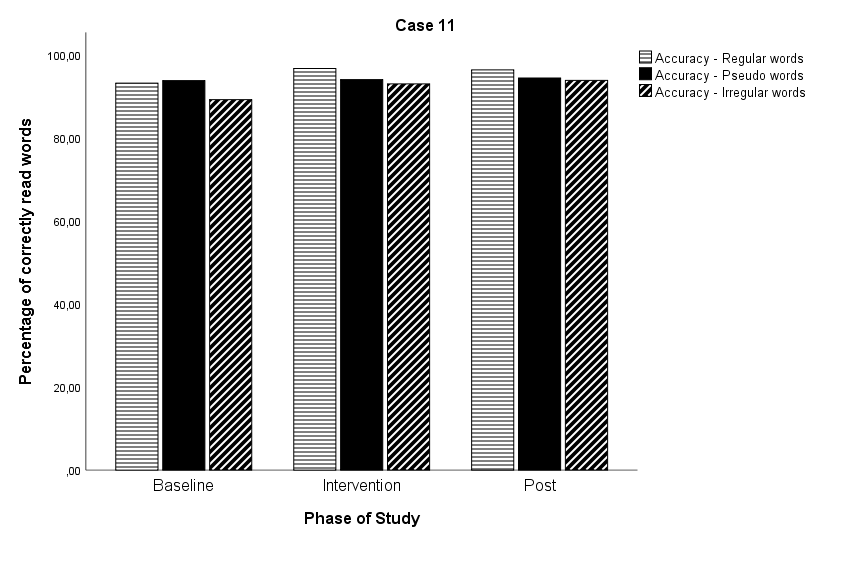


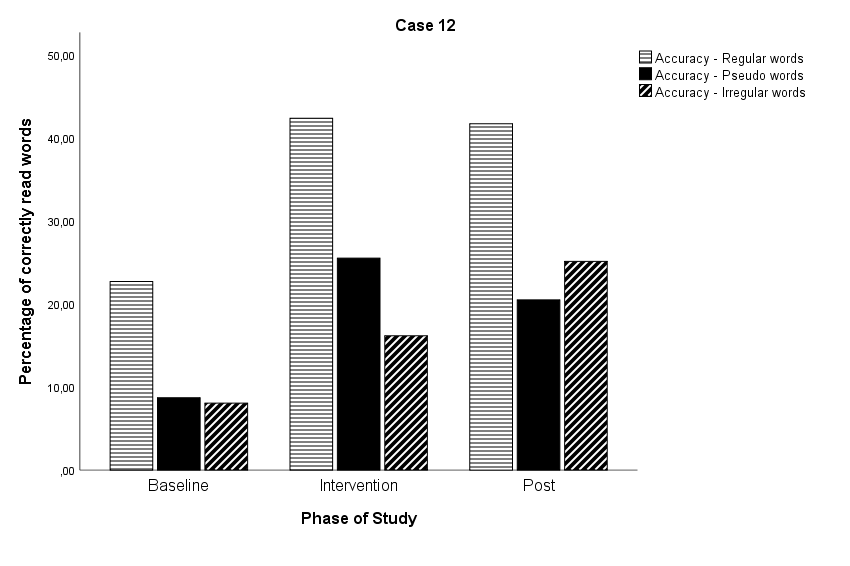


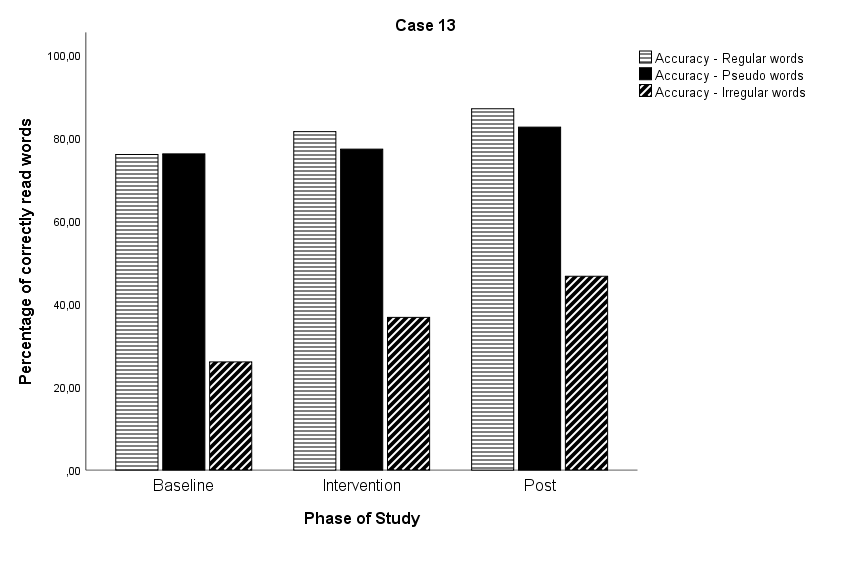


**Supplemental material**

**Intervention programme – Guidelines for Teachers**

**Introduction**

The purpose of the training is to assess whether students with dyslexia can benefit from articulatory consciousness training. Many students with reading, writing and language disorders have problems discriminating speech sounds through listening, thereby disturbing the process of sound/sign mapping in reading. Through the training, we seek to assess whether learning to define phonemes by their articulatory features – that is, how different parts of the speech organ are activated when pronouncing a sound – can increase decoding efficiency.

Phonemes can be categorised according to how they are pronounced: Where do you place your tongue? What about the airflow? Is the sound voiced or not? The basic symbols in PAS indicate how the articulatory system works when pronouncing different sounds. Each PAS card contains information about how to pronounce the sound. Together, the elements on the cards constitute the way in which to correctly pronounce the sound. Each card contains ‘the recipe’ for the sound presented.

The purpose of the training is to learn how to interpret symbols, become aware of what to do with the tongue and voice when pronouncing sounds and learn how to utilise this as support in reading and writing. The student is not supposed to remember the symbols as visual units. Instead, the focus should be on following the ‘recipe’ to produce the correct sound. Each session is 40 minutes long and consists of a set of four to five activities, each of which is five to ten minutes long.

**General guidelines**

**The role of the teacher**

Prior to the intervention onset, the teacher should gain some knowledge about the PAS system, but gaining expertise is not a requirement. The teacher’s attitude during the training should be collaborative so that, together, the teacher and student can explore the system using the ‘secret’ signs. The teacher’s role is mainly to model how the sounds are made with the mouth and help the student draw attention to the information contained in the symbols.

When the work starts for the first time, the teacher may introduce the training in this way: ‘For those who have dyslexia, it is difficult to read and spell. Now, we will try to read and spell using another system; it will be almost like learning secret signs…’

**Progression**

The recommendation is to first start with a selection of PAS symbols and then gradually introduce new symbols. The basic symbols are those for I, S, M, O and L.

When to introduce new symbols depends on how quickly the student learns the PAS principles and the student’s progress in learning the basic symbols. (According to prior experience, this is often less problematic for students compared to teachers!)

**Log form**

A log form has to be completed after each session to confirm that the programme has been followed. However, the teacher can also use the log to enter comments regarding the student’s motivation, progress, use of strategies, etc.

**Intervention materials**

- Several copies of each PAS symbol, with the corresponding letter on the back, printed on stiff paper and laminated
- A3 poster picturing all PAS symbols

**Additional materials**

- Picture cards. Picture cards. For instance, use cards from memory games if this is available at school.
- Other sorts of pictures, e.g. from newspapers, magazines and downloads from the Internet.
- Mirrors

Texts on the right reading level

**Content of activities**

**1. Learning the basic elements of the PAS symbols**

The student and teacher investigate the basic pictographic elements of the PAS symbols together. For each session, they repeat what these elements mean and which parts of the articulatory system they refer to. In this session, the student and teacher can also sort the cards by categorising them, e.g. ‘All cards with an indicator for voice on’, ‘all nasals’, all with a ‘whisper sound’, etc. A mirror is recommended to make this exercise more concrete.

**2. Matching PAS symbols to alphabetic letters**

The student and teacher sit at the table facing each other. Selected PAS cards are stacked in a bunk with the symbol facing upside. The student’s role is to draw a card, analyse the elements and pronounce the sound. To help the teacher, the PAS “graphemes/ letters ” are written in alphabetic script on the back of the card. The teacher is to scaffold/support the student when needed, but the student is not allowed to see “the facet” until the sound is pronounced.

Afterwards, the cards are stacked with the alphabetic script (the facet) facing upside. The student draws a card and then matches it to the correct PAS symbol by putting it in the correct place on a ‘PAS overview poster’ containing all the pictographic symbols.

**3. Spelling words by PAS cards**

The student spells a word using the PAS cards, and the teacher reads. The teacher can also spell a word, with the student reading. Picture cards, for instance from children`s matching- and memory games can be used: The PAS cards must lay upside on the table. The student draws a picture card, says the word out loud and then matches this card to the PAS symbol from the first (or last) sound in the word. Alternatively, the student can draw a card and spell the word using the PAS cards.

Working with “minimal pairs”: The purpose is to increase the student’s phonological awareness by helping them become aware that a single phoneme can change the meaning of a words, e.g. hat-cat, seal-meal, etc. The student closes their eyes, and the teacher puts up (“spells”) words with the PAS cards. The student then opens their eyes and reads the word. Thereafter, the student again closes their eyes, and the teacher changes the word by replacing one card. As a variation, the student may spell the words with the articulatory cards, with the teacher closing her eyes and reading.

**4. Text-reading exercise**

The teacher selects a text from the student’s reading level, which the student reads out loud, and the teacher marks any words read with effort. The student works with the details of the phonological structure of these words by using the PAS cards to spell the words. The teacher encourages the student to use audible or silent articulation as a support. Afterwards, the student re-reads the same text and is also encouraged to articulate the sounds clearly during the text reading.

***Activity 4 should not be introduced until the student has become familiar with all the symbols in PAS.***

**5. Sort the cards and put them back in the box**

The student is to sort the cards in alphabetic order and put them back in the container.

NB: The time recommendations for the activities are indicative and may be adjusted gradually. For example, it is likely that, after some weeks of training, activities 1 and 5 will require less time than activities 2 and 3. However, due to the need for variety, the activities should change during each session.

The form is the teacher's assessment of the student's motivation and efforts in the special education sessions both prior to intervention and during the intervention period. There are also questions concerning your evaluation of the student's benefit from the intervention. All questions are answered by ticking the table below. The table uses a scale from 1 to 6 where 1 is the lowest negative value and 6 is the highest positive value. There is also space for your own comments at the bottom!

1. **The student's motivation for School Work in general.**

| 1 | 2 | 3 | 4 | 5 | 6 |
| --- | --- | --- | --- | --- | --- |
|  |  |  |  |  |  |

1. **The student's efforts in School Work in general.**

| 1 | 2 | 3 | 4 | 5 | 6 |
| --- | --- | --- | --- | --- | --- |
|  |  |  |  |  |  |

1. **Frequency of school absence.**

| 1 | 2 | 3 | 4 | 5 | 6 |
| --- | --- | --- | --- | --- | --- |
|  |  |  |  |  |  |

1. **The student`s general motivation for reading and writing (prior to intervention).**

| 1 | 2 | 3 | 4 | 5 | 6 |
| --- | --- | --- | --- | --- | --- |
|  |  |  |  |  |  |

1. **The student`s effort in working with reading and writing (prior to intervention).**

| 1 | 2 | 3 | 4 | 5 | 6 |
| --- | --- | --- | --- | --- | --- |
|  |  |  |  |  |  |

1. **The student`s general motivation for special needs education (prior to intervention)**

| 1 | 2 | 3 | 4 | 5 | 6 |
| --- | --- | --- | --- | --- | --- |
|  |  |  |  |  |  |

1. **The student`s effort in the special education sessions (prior to intervention).**

| 1 | 2 | 3 | 4 | 5 | 6 |
| --- | --- | --- | --- | --- | --- |
|  |  |  |  |  |  |

1. **The student`s general motivation to participate in the project.**

| 1 | 2 | 3 | 4 | 5 | 6 |
| --- | --- | --- | --- | --- | --- |
|  |  |  |  |  |  |

1. **The student`s effort during the intervention period.**

| 1 | 2 | 3 | 4 | 5 | 6 |
| --- | --- | --- | --- | --- | --- |
|  |  |  |  |  |  |

1. **The student`s effort in the sessions during the intervention period compared to the effort in previous special education sessions.**

| Much poorer | Poorer | Slightly poorer | As before | Slightly better | Better | Much  better |
| --- | --- | --- | --- | --- | --- | --- |
|  |  |  |  |  |  |  |

1. **General effort during the intervention period.**

| Evenly poor throughout the period. | Most effort from the start, then decreasing. | Little effort from the start, then increasing. | Consistently good throughout the period. |
| --- | --- | --- | --- |
|  |  |  |  |

1. **General benefit of the training with the PAS cards?**

| 1 | 2 | 3 | 4 | 5 | 6 |
| --- | --- | --- | --- | --- | --- |
|  |  |  |  |  |  |

1. **To what extent has the training with the PAS cards led to a change in the student's reading strategy?**

| 1 | 2 | 3 | 4 | 5 | 6 |
| --- | --- | --- | --- | --- | --- |
|  |  |  |  |  |  |

1. **To what extent has the training with the PAS cards led to increased reading accuracy?**

| 1 | 2 |  | 3 | 4 | 5 | 6 |
| --- | --- | --- | --- | --- | --- | --- |
|  |  |  |  |  |  |  |

1. **To what extent has the training with the PAS cards led to increased reading fluency?**

| 1 | 2 | 3 | 4 | 5 | 6 |
| --- | --- | --- | --- | --- | --- |
|  |  |  |  |  |  |

1. **To what extent has the training with the PAS cards led to decreased rate of spelling errors?**

| 1 | 2 | 3 | 4 | 5 | 6 |
| --- | --- | --- | --- | --- | --- |
|  |  |  |  |  |  |

1. **Has more than one teacher participated in the teaching with the PAS cards? Tick your answer.**

**No Yes**

**If so, indicate how many teachers and in how many sessions.**

**………………………………………..**

**Other comments:**

| **If necessary, use the next page☺** |
| --- |
